# From Structure to Strength: Analyzing the Impact of Sulfuric Acid on Pig Bone Demineralization Through FTIR, LIBS, and AAS

**DOI:** 10.3390/ijms252212250

**Published:** 2024-11-14

**Authors:** Milica Marković, Miroslav Kuzmanović, Dragan Ranković, Danica Bajuk-Bogdanović, Aleksandra Šajić, Dušan Dimić

**Affiliations:** 1 Faculty of Physical Chemistry, University of Belgrade, Studentski Trg 12-16, 11000 Belgrade, Serbia; milica.markovic@ffh.bg.ac.rs (M.M.); miroslav@ffh.bg.ac.rs (M.K.); danabb@ffh.bg.ac.rs (D.B.-B.); aleksandra.sajic@ffh.bg.ac.rs (A.Š.); 2 Vinča Institute of Nuclear Sciences-National Institute of the Republic of Serbia, University of Belgrade, Mike Petrovića Alasa 12-14, 11000 Belgrade, Serbia; ranko@ffh.bg.ac.rs

**Keywords:** pig bone, sulfuric acid, demineralization, collagen/phosphate content, collagen maturity, ATR-FTIR, LIBS, AAS

## Abstract

The present research aimed to investigate the demineralizing effects of sulfuric acid on pig bone. Alterations in collagen and phosphate contents and changes in the elemental composition of the bone during the 14-day-long immersion in sulfuric acid solutions of different concentrations were estimated using ATR-FTIR, LIBS, and AAS. FTIR spectra at amide I (1800–1600 cm^−1^) and phosphate ν_1_/ν_3_ (PO_4_^3−^) (1300–900 cm^−1^) domains were scrutinized using the deconvolution method for monitoring changes in the protein secondary structure and mineral content. The results implicated sulfuric acid as a powerful demineralization agent and effective in targeting mineral components, such as hydroxyapatite, while leaving the collagen matrix relatively preserved with a complex secondary structure. Collagen maturity marker values gave valuable insights into the structural integrity of the bone. LIBS and AAS indicated changes in bone hardness; phosphorous-to-carbon ratio; and calcium, phosphorous, and magnesium content in the solutions left after the immersion period. The changes in the ratio of ionic-to-atomic calcium lines in the LIBS spectra indicated hardening of the bone, with increasing acid concentration and prolonged action, due to the deposition of calcium sulfate on the surface. The calcium concentration in the solutions decreased with increased acid concentration, while the change in phosphorus and magnesium concentrations was reversed.

## 1. Introduction

Animal bones are usually found in archaeological, anthropological, and forensic sites. Their interdisciplinary significance is reflected in the complex information they give, from domestication and hunting practices over climatic conditions and evolutionary processes to taphonomic processes and trauma investigations. Due to biological and anatomical similarities to human bones, in terms of lamellar bone structure, rate of bone regeneration, cortical bone mineralization rate, concentration of bone mineral, and bone mineral density, pig bones have an important role across all these scientific areas [1,2,3].

Sulfuric acid is one of the most dangerous acids; it is a very corrosive, reactive, and dehydrating agent, causing different degrees of damage depending on how it comes into contact with biological tissue (dermal exposure, ingestion, or inhalation). From an archeological standpoint, the effect of sulfuric acid from natural (mineral oxidation, volcanic activity, decomposition of organic matter, and acid rain) and anthropogenic sources (pollution) is significant as it influences the preservation of bone material, causing the loss of details like sex and age characteristics. In forensic pathology practice, it is very often an agent used to destroy physical evidence of biological origin, disable determination of the cause and time of death, and to impede victim identification [1,2,3].

The skeleton of bone tissue is the main supporting structure of the vertebrate body, which consists of bones and cartilage. Muscles, tendons, and ligaments are also attached to bones and cartilage. The skeleton provides the rigidity of the body and a series of mechanical levers to which the tissues are attached and allows the movement of the muscles and parts/whole of the body [4,5].

The structure of the bone provides an ideal balance of hardness and elasticity. Bone is hard enough to withstand external forces, although poorly mineralized bone is brittle and prone to fractures. At the same time, the bone must be light enough to move when the muscles contract. Two-thirds of bone weight is in minerals, and the rest is in water and mostly in type I collagen. Collagen is a fibrous structural protein in animals’ extracellular matrix and connective tissue. It is the most abundant protein in the animal kingdom, but it is absent in the tissue of plants and unicellular organisms, where polysaccharides and cellulose take over its role. Collagen makes up 25–30% of the protein content of the whole body, especially in mammals, where it enters the structure of the blood vessels, cornea, bones, cartilage, tooth dentine, etc. [4,5,6]. The basic structural unit of collagen is heteropolymer, which consists of three polypeptide chains arranged as a triple helix. The collagen structure has a repeating Gli-X-Y motif of the amino acid glycine and, most often, the amino acids proline and hydroxyproline. Collagen fibers are “binding sites” for calcium and other bone minerals, and this process is known as bone mineralization. Non-collagen matrix proteins include proteoglycans, proteins containing γ-carboxyglutamate, glycoprotein osteonectin, phosphoprotein osteopontin, and growth factors. There is also a small amount of lipids in the bone tissue [4,5,6].

Hydroxyapatite, Ca_5_(PO_4_)_3_OH (HA) (usually written as Ca_10_(PO_4_)_6_(OH)_2_ to emphasize that the unit cell consists of two structural entities), is the mineral from the group of calcium phosphates, which in terms of chemical, structural, and morphological characteristics best corresponds to natural bone tissue. Calcium phosphates are salts of phosphoric acid whose compounds contain H_2_PO_4_^−^, HPO_4_^2−^, and PO_4_^3−^ ions in their structure. Biological apatites contain two inorganic orthophosphate ions, PO_4_^3−^ and HPO_4_^2^, as part of bones, tooth enamel, and various pathological calcifications. Biological apatites are structurally changed hydroxyapatite, non-stoichiometric apatites, usually deficient in calcium and always with an incorporated carbonate group, CO_3_^2−^. Biological apatites are made of small apatite crystals, which is a significant factor when discussing the solubility of biological apatites compared to mineral apatites. Small dimensions and low crystallinity are two pronounced features of biological apatites, which, in combination with non-stoichiometric composition, internal crystal disorder, and the presence of carbonate ions in the lattice, explain its unique characteristics. Apatite structures allow large deviations in composition, with the ability to accommodate a large number of different anionic (e.g., CO_3_^2−^ (up to 5%), HPO_4_^2−^ (up to 5–10%), Cl^−^), and cationic (e.g., Na^+^, Mg^2+^, K^+^) substituents in their three sublattices. There are several substitution patterns in bone: B type, where CO_3_^2−^ ions can take the place of PO_4_^3−^ ions; A type, where CO_3_^2−^ ions can take the place of OH^−^ ions; and substitution, when mono hydrogen-phosphate ions, HPO_4_^2−^, take the place of PO_4_^3−^ ions [7,8].

The most important analytical tool used to investigate bones is spectroscopic methods. In this paper, the structural aspects of the bone organic and mineral matrix are determined by Attenuated Total Reflectance–Fourier Transform Infrared spectroscopy (ATR-FTIR), while the changes in the elemental composition of the bone surface are examined by Laser-Induced Breakdown Spectroscopy (LIBS). The elemental composition of the solution remaining after the extraction of the bone was determined by atomic absorption spectrometry (AAS).

FTIR is considered the most common in bone analysis as a non-destructive, rapid, and specific vibrational spectroscopic method [9,10,11,12,13,14,15,16,17,18,19,20,21,22]. FTIR spectroscopy has been extensively applied in bone tissue analysis, giving insight into the tendon-to-bone or ligament-to-bone interfaces and different tissue transitions. Recent advances in the development of FTIR confirm the promising potential for its application in forensic biological analysis. The most advanced modalities, including hyperspectral imaging (the coupling of spectrometers to imaging systems) and fiber optic probes, combined with chemometrics, have been proven invaluable in obtaining complete and reliable results [23].

LIBS is an in situ method based on OES, Optical Emission Spectroscopy. LIBS has demonstrated its unique versatility as an analytical technique, enabling fast, non-contact analyses of almost all types of materials and the ability to adapt the method to the unique requirements of various practical analytical problems. The main advantages of the LIBS technique are the ability to analyze all aggregate states, test biological samples, and test samples at high temperatures and pressures and detect almost all elements simultaneously. The LIBS technique has become a powerful tool in fundamental studies of laser interactions with different materials. The method is applicable in analyzing forensic samples and bones, providing the possibility of working in situ on small bone fragments and preserving them for further analysis when necessary [24,25,26,27,28,29].

The phenomenon of atomic absorption has been known since the beginning of the 19th century. AAS involves several techniques that provide quantitative information about atoms independently of the molecular forms in which they are found in a sample. For the high-precision elemental analysis of bones, it is necessary to convert the sample into a solution by digestion using mineral acids. One of the very convenient and widely available analytical techniques is flame atomic absorption spectrometry (FAAS). FAAS is one of the most frequently used tools in analytical chemistry, including forensic analytical analysis [30].

The results of this work provide quantitative insight into the effects of different concentrations (0.01, 0.1, and 1 M) of aqueous sulfuric acid solutions on pig shoulder bone after the immersion periods of 1, 7, and 14 days. Special attention is paid to the changes in the secondary structure of collagen and phosphate content of the bone upon the action of acid.

## 2. Results and Discussion

**Figure 1:**
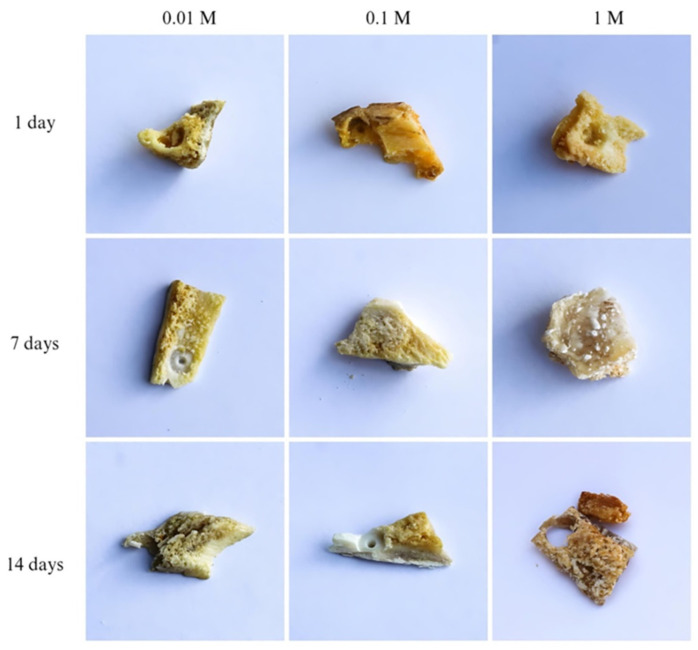
Pig shoulder bone after immersion in sulfuric acid.

**Figure 2:**
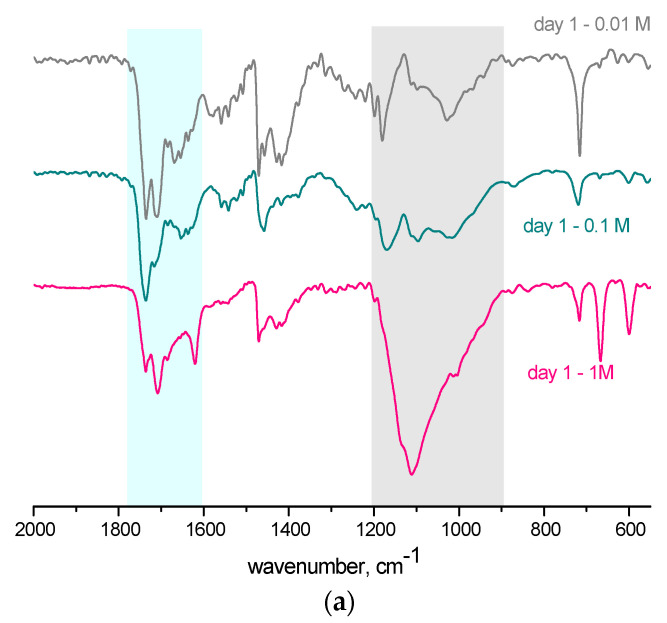

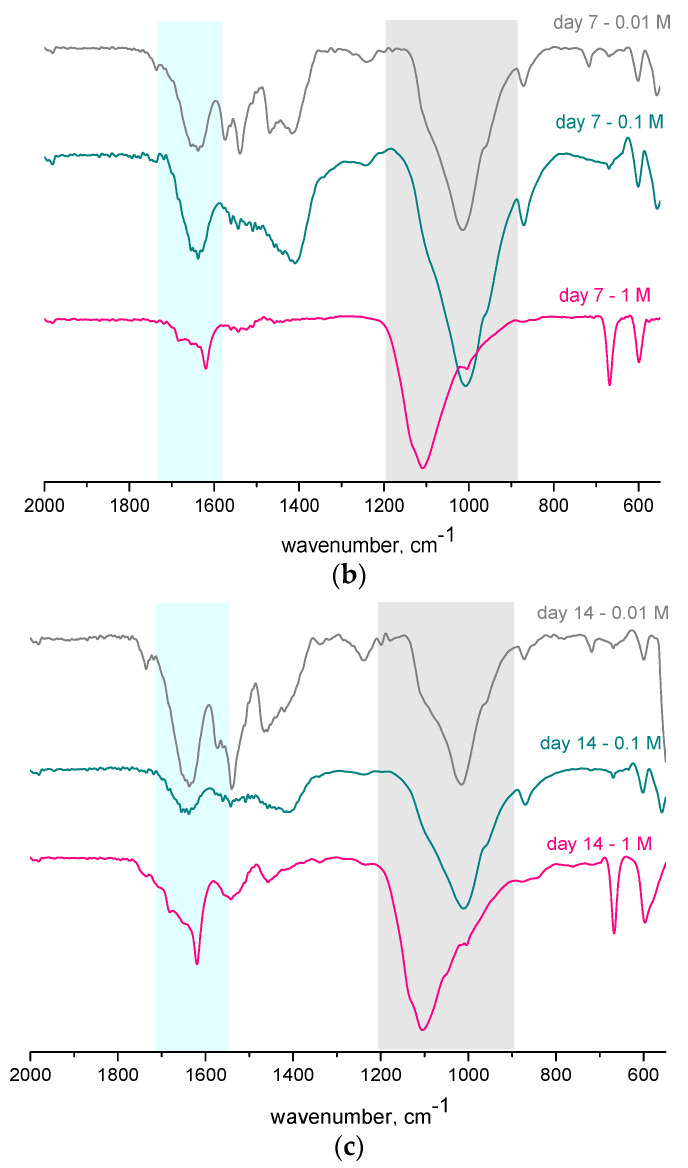
Comparative display of ATR–FTIR spectra of pig shoulder bone in sulfuric acid (0.01, 0.1, and 1.0 M) after immersion periods of 1 (**a**), 7 (**b**), and 14 (**c**) days.

The analyzed bone samples were all obtained by cutting pieces of the same bone with approximately the same mass, color, and visual structure. Generally, immersing bone in acids causes the hydrolysis of proteins and the degradation of bone apatite. Starting from the visual changes in the bone (Figure 1), the influence of sulfuric acid is monitored through changes in the structure of the bands assigned to the vibrational modes of the organic (amide I region) and inorganic phases (ν_1_/ν_3_ (PO_4_^3−^) domain) of the bone (Figure 2).

### 2.1. Amide I Domain

The amide I band positioned between 1800 and1600 cm^−1^ (Figure 2 and Figure S1) is assigned to stretching vibrations of the C=O (70–85%) and C-N (10–20%) bonds in the peptide backbone of proteins. The structure and position of the amide I band are susceptible to alternations in the surrounding medium, such as heat, moisture, or chemicals, including acids [31]. The conformation stability of collagen is dependent upon intrachain forces along the polypeptide chain.

The secondary structure of collagen is compromised by the action of mineral acids, which break down hydrogen bonds and other non-covalent interactions in its structure [31]. Significant changes in the intensity (generally very low, 71–93% T), position, and structure of the amide I band are observed with increasing acid concentration and the time of acid action (Figure 2). In the FTIR spectra shown in absorption (Figure S2), it is evident that the amide I band is very complex and even extends above 1700 cm^−1^ in all spectra recorded after the first 24 h of acid action. As the immersion period prolongs, the specific features of the bands in the 1800–1700 cm^−1^ region practically disappear, or drastically decrease in intensity, from the spectra recorded on the seventh and fourteenth days.

Deconvolution in the amide I region (Figures S3–S6) demonstrates a complex secondary structure of the collagen matrix dependent upon acid concentration and immersion time. Table 1 lists the results of the curve fitting analysis and sub-band assignment performed according to literature data [32,33,34,35,36]. As Table 1 depicts, various conformations of the collagen secondary structure are observed: triple helix, β-turn, α-helix, parallel β-sheet, unordered structure, pyridinoline cross-links, anti-parallel β-sheet, DHLNL, and HLNL cross-links. Moreover, additional sub-bands assigned to different amino acid side chains, tyrosine, arginine, and glutamic acid side chains, are also present.

As evident from Table S1, the primary forms of the secondary collagen structure remain in the spectra of the reference sample (Figure S3). A slight reduction in integrated areas of the bands assigned to the triple helical and β-turn structures, caused by prolonged exposure, can result from hydration and the loss of structural integrity. In contrast, an increasing trend in the integrated areas of the sub-bands assigned to the triple helix (~1639 cm^−1^) and β-turn (~1609 and 1618 cm^−1^), with increasing immersion time and acid concentration, is observed (Table 1 and Table S1). The obtained result leads to the conclusion that the main form of the secondary structure of collagen, the triple helix, is preserved as mineral dissolution progresses.

The preservation of the triple helix structure, a hallmark of collagen’s mechanical properties, implies that, despite the acid-induced challenges, the primary structural motif vital for maintaining tensile strength may still be preserved. This suggests that some of the triple helix structures could be stabilized due to reorganization or cross-linking and that the overall mechanical integrity of the bone might somewhat withstand the acid exposure. However, other mechanical properties, such as compressive strength or toughness, could not be guaranteed by the persistence of these collagen conformations. The bone’s long-term structural integrity may still be compromised by the ongoing degradation processes, including hydrolysis and oxidation triggered by sulfuric acid, potentially leading to increased brittleness and reduced resilience as mineral components dissolve. The obtained results have potential forensic implications as the consistent presence of these sub-bands across varying acid concentrations and immersion periods could indicate how long bones have been exposed to such environments, potentially helping forensic analysis in establishing the timelines or identifying specific treatments the remains may have undergone. This knowledge could be important for interpreting the preservation state of the bone and could aid in identifying scenarios of chemical exposure or intentional degradation.

Degradation of collagen is caused by oxidation, hydrolysis, and denaturation. Sulfuric acid is a potent hydrolyzing and oxidizing agent, and as a consequence of oxidation, besides the presence of the sub-bands assigned to different forms of the secondary structure of collagen, in the deconvoluted spectra, the bands assigned to carbonyl compounds (~1730–1740 cm^−1^), which are formed during the fragmentation of polypeptides, are also evident [37,38]. The relative area of the band assigned to the carbonyl vibrations in fragmented polypeptide chains is the biggest during the first 24 h of immersion when all the solutions are considered. After the first 24 h, the relative area of this band decreases, which most likely indicates further transformation of the formed carbonyls.

The analysis in the amide I domain is focused primarily on two underlying sub-bands, around 1660 and 1690 cm^−1^, assigned to nonreducible (mature) (Pyr, Pyridinoline) (~1660 cm^−1^) and reducible (immature) (DHLNL, Dihydroxynorleucine) (~1690 cm^−1^) collagen cross-links. The area ratio of these bands is referred to as collagen maturity marker (CMM). It is related to the cross-linking of collagen fibers, one of the most prominent features of type I collagen in mineralized bone tissue [31,39,40,41,42]. Collagen cross-links are important for tensile strength and viscoelasticity of the bone. This characteristic is prone to changes caused by certain diseases, aging, overall bone health, external factors like burial environment, or the effect of chemicals. As Table 1 and Table S1 show, an increasing trend in the integrated area of the sub-band assigned to mature pyridinoline collagen cross-links is evident, while the area of the band assigned to immature DHLNL and HLNL cross-links remains almost unchanged (between 12.0–12.6%). It is important to note that in 0.1 M solution after 7 days of immersion, a band assigned to deoxypyridinoline cross-link is present (1681 cm^−1^), unlike other solutions.

**Figure 3:**
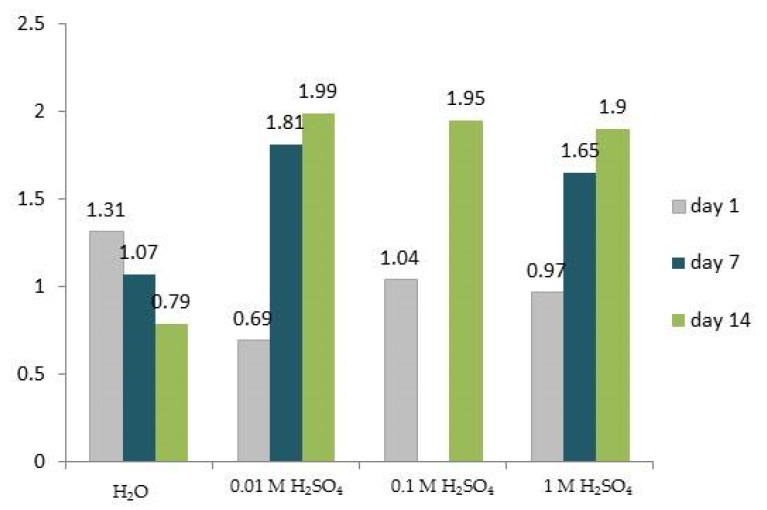
Collagen maturity marker values are calculated as the area ratio of the sub-bands at 1660 and 1690 cm^−1^.

The integrity of cross-links within the collagen matrix is affected by harsh chemical treatment. Figure 3 presents the change in CMM values. In reference samples, as the immersion time prolongs, the CMM decreases, which can be explained by initial hydration and swelling of collagen, which can alter the spacing between collagen fibers and affect the structure and stability of the cross-links. In sulfuric acid maturity, marker values for each concentration shift to a higher proportion of mature cross-links with more extended acid action, representing “bone aging”. A possible explanation could be the breakdown of non-collagenous proteins that may inhibit collagen cross-linking. As sulfuric acid concentration increases and exposure time prolongs, removing non-collagenous components is more extensive, leading to advanced cross-linking within collagen molecules. Generally, the increasing trend in CMM is the aspect of bone remodeling in vivo, where a higher proportion of mature cross-links is generally characteristic of older bones, influenced by already mentioned factors, which are also in agreement with biochemical analyses [31]. Nevertheless, it is important to understand that these alterations are primarily a result of the harsh chemical conditions applied during the experiment rather than the natural dynamics of bone remodeling seen in physiological or pathological conditions and typical biological aging. If individual days are analyzed, the CMM changes between 5 and 30% with increasing acid concentration and ranges from 0.69 to 1.04 (day 1), from 1.81 to 1.65 (day 7), and from 1.90 to 1.99 (day 14). The result indicates that the efficiency of the acid is more pronounced in more concentrated solutions [38].

### 2.2. Phosphate, 1300–900 cm^−1^, ν_1_/ν_3_ (PO_4_^3−^) Domain

Bands assigned to four vibrational modes of biological apatites (ν_1_–ν_4_), which contain two inorganic orthophosphate ions, PO_4_^3−^ and HPO_4_^2−^, occur in three distinct parts of the mid-IR region of the spectrum. Due to the tetrahedral symmetry (Td) of the free phosphate ion, the ν_1_ (A1) (symmetric stretching, ~960 cm^−1^) and ν_2_ (E) (symmetric bending, ~473 cm^−1^) modes are IR-inactive. When the crystal symmetry is lowered, the ν_1_ mode becomes IR-active and appears as a weak band between 950 and 970 cm^−1^. In poorly crystalline apatites, the band assigned to this mode is usually visible as a shoulder of ν_3_ asymmetric stretching modes of the HA. The vibrational mode ν_3_ of the HA is triple-degenerate and very sensitive to the environment. These two modes together give a wide band, usually designated as ν_1_/ν_3_ (PO_4_^3−^), in the 1300–900 cm^−1^ region. A fundamental ν_4_ (PO_4_^3−^) bending mode appears as a complex band in the 650–500 cm^−1^ region [13].

The dynamic nature of apatitic crystals is reflected in the band’s structure, intensity, and shape assigned to ν_1_/ν_3_ (PO_4_^3−^). As evident from Figure 2, the band in the 1300–900 cm^−1^ region is wide and intensive, indicating a complex structure composed of multiple sub-bands. The intensity of the band varies between 78 and 92%T (0.01 M), 78 and 85%T (0.1 M), and 57 and 78%T (1 M).

In the FTIR spectra of the reference sample (Figure S3), there are no significant changes in the structure and position of bands, only a decrease in intensity over time. FTIR spectra presented in absorption (Figures S7 and S8) show that the ν_1_/ν_3_ (PO_4_^3−^) band undergoes significant changes in intensity, structure, and position with increasing acid concentration. By increasing acid concentration for every period of immersion, the band undergoes a substantial shift towards higher energies, up to 102 cm^−1^, and a significant increase in intensity. During the first day of acid action in the spectra of 0.01 and 0.1 M solutions, the ν_1_/ν_3_ (PO_4_^3−^) band is extremely wide, expanding above 1200 cm^−1^. With increasing acid concentration, the intensity of the band increases, and the bands above 1200 cm^−1^, characteristic of the amide III region, disappear. As the time of acid action is prolonged (7 and 14 days), the (PO_4_^3−^) band in the spectra of 0.0.1 and 0.1 M solutions is still wide but of a changed shape and structure (Figure S8b,c) compared to the spectra of the exact solutions after 24 h (Figure S8a). After more prolonged exposure, 7 and 14 days, the band’s intensity increases significantly with increasing acid concentration. A significant increase in the intensity of the ν_1_/ν_3_ (PO_4_^3−^) phosphate band can certainly be the result of the formation of calcium sulfate (the region between 1100 and 1150 cm^−1^ is typical of the sulfate group in the calcium sulfate, Figure S9), as further examined by LIBS and AAS.

**Figure 4:**
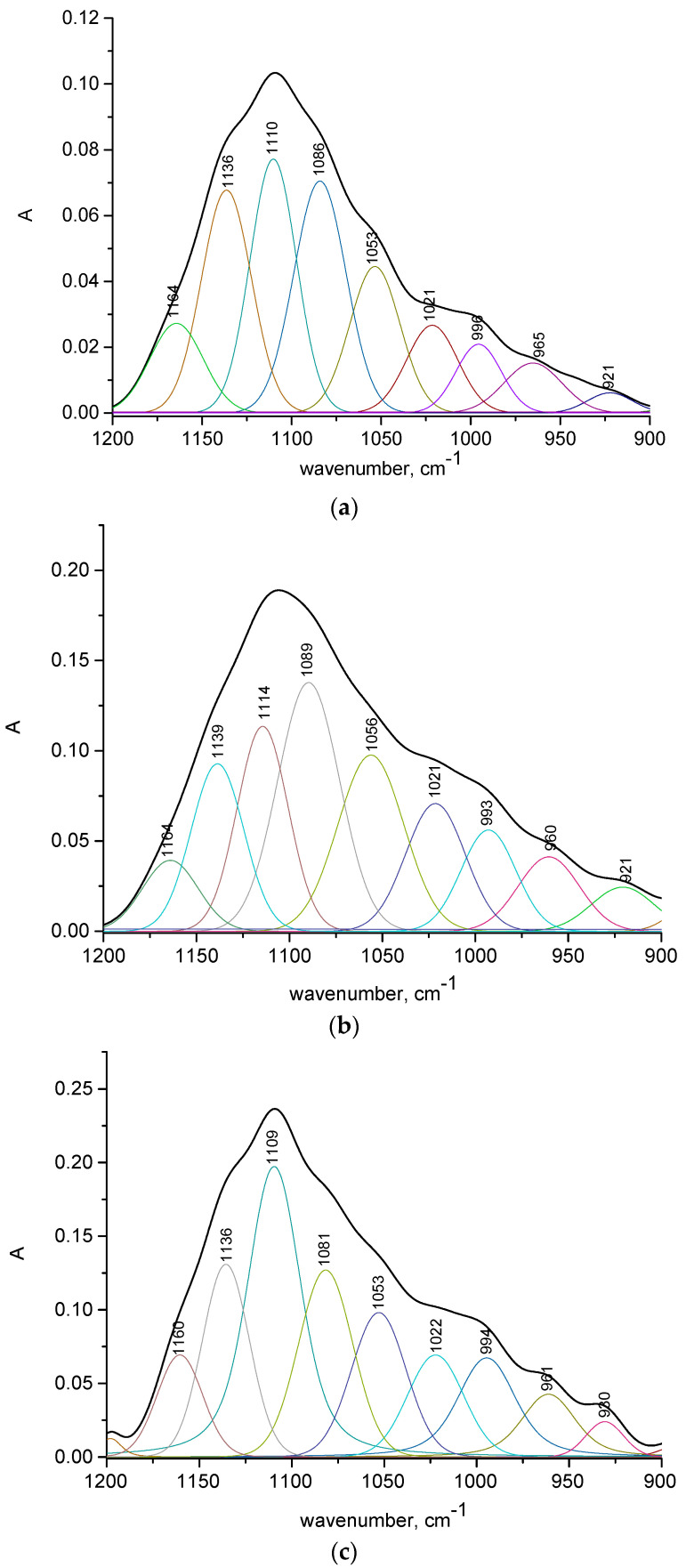
The results of the curve fitting analysis in the 1200–900 cm^−1^ IR region of pig bone in 1.0 M sulfuric acid: immersion time, 1 (**a**), 7 (**b**), and 14 (**c**) days.

Table S2 and Figure 4, Figures S10 and S11 show the results of the curve fitting analysis in the phosphate 1300–900 cm^−1^ region. After deconvolution, multiple peak positions were identified and assigned to specific vibrational modes, orthophosphate ion, non-stoichiometric HA-containing vacancies, HPO_4_^2−^ and/or CO_3_^2−^, ν_3_ (T2) vibrational modes of apatite, and the domain of poorly crystalline apatites [14,15,16,17,18,19].

The deconvolution spectra of reference solutions (Table S2, Figure S3) are dominated by sub-bands assigned to HPO_4_^2−^-PO symmetric stretch, ν_1_ (PO_4_^3−^) in apatitic/nonapatitic environment, non-stoichiometric hydroxyapatite containing vacancies (HPO_4_^2−^ and/or CO_3_^2−^), type B carbonate hydroxyapatite, and ν_3_ (T_2_) vibrational modes of apatite. The greatest integrated area has a band assigned to ν_1_ (PO_4_^3−^) vibration in an apatitic environment, which slowly decreases with exposure time (28.9, 20.3, and 19.7%, respectively). On the contrary, the integrated areas of the band assigned to HPO_4_^2−^-PO symmetric stretch and non-stoichiometric hydroxyapatite containing vacancies (HPO_4_^2−^ and/or CO_3_^2−^) increase with time of exposure (13.5, 25.8, and 26.1 and 18.2, 24.9, and 26.5, respectively). Non-stoichiometric hydroxyapatite often has vacancies or defects, which can accommodate additional anions like HPO_4_^2−^ and CO_3_^2−^ as the mineral phase is altered by acid exposure. The incorporation of these ions can contribute to the intensity of the FTIR bands associated with these species.

The deconvoluted spectra of 0.01 and 0.1 M solutions (1 day of immersion) are very complex, with multiple sub-bands that extend even to the amide III region (1300–1200 cm^−1^) (Table S2, Figures S10 and S11). The integrated area of the band assigned to ν_1_ (PO_4_^3−^) symmetric stretch vibration varies between 5.0 and 14.4% and is somewhat lower in more concentrated solutions (1 M) (Table S2). The band assigned to ν_3_ (PO_4_^3−^) (~1030 cm^−1^) in well-crystallized stoichiometric apatites is present only in the deconvoluted spectrum of 0.01 M solution after 24 h of immersion. This band is shifted to 1020 cm^−1^ with prolonged acid action, indicating non-stoichiometric apatite containing vacancies, HPO_4_^2−^, and/or CO_3_^2−^. In the deconvoluted spectra of the 0.01 (days 7 and 14) and 0.1 M (days 1–14) solutions (Table S2, Figures S10 and S11), the band around 1020 cm^−1^ is the most intense, with integrated areas between 12.7 and 26.5%. In the spectra of the 0.01 and 0.1 M solutions, the bands assigned to the ν_3_ (T2) vibrational modes of apatite are also relatively intense (Table S2, Figures S10 and S11).

In 1 M solutions, for all time intervals of acid action, the band assigned to non-stoichiometric apatite (~1020 cm^−1^) has an integrated area between 7.8 and 10.7%, which is less intense compared to the spectra of the 0.01 and 0.1 M solutions. The bands assigned to the ν_3_ (PO_4_^3−^) domain in stoichiometric apatites (~1090 cm^−1^, integrated area between 21.0–21.8%) and the ν_3_ (PO_4_^3−^) domain of poorly crystalline apatites (~1110 cm^−1^, integrated area between 14.8 and 26.9%) become more intense compared to the 0.01 (4.5% and 1.8–10.9%, respectively) and 0.1 M solutions (10.8–14.7% and 4.3–8.2, respectively) [16,17].

It is important to note that the quantitative parameters, as a measure of chemically induced alterations in bone, like the mineral-to-matrix ratio, could not be calculated from FTIR data due to the complexity of the amide I region, fragmentation of polypeptides, the overlapping of phosphate and sulfate bands (from formed calcium sulfate), and even the amide III band. The complexity of the amide I region in FTIR spectra is a major factor affecting the reliability of quantitative measures in studies involving the mineral-to-matrix ratio. The amide I band, primarily arising from C=O stretching vibrations of the peptide bonds within collagen, typically appears around 1650 cm^−1^. However, the potential overlaps, originating from the fragmentation of polypeptides; overlapping phosphate and sulfate bands; and amide III band interference can obscure the distinct amide I band, making it challenging to isolate collagen contributions from those of hydroxyapatite or other minerals that may be altered during acid treatment. Fragmentation of collagen polypeptides results in shorter peptides that may produce additional or shifted spectral features. This fragmentation can contribute to a broadening of bands and the emergence of new absorption bands in the amide I region, making it difficult to discern the contribution of intact collagen versus decomposed fragments. Moreover, the presence of varying degrees of fragmentation can also distort the intensity of the amide I band, complicating the determination of the mineral-to-matrix ratio. Additionally, treating the bone with sulfuric acid induces calcium sulfate formation, which can introduce additional bands into the FTIR spectrum. The phosphate bands (typically present around 1200–900 cm^−1^ and associated with hydroxyapatite) overlap with spectral features of sulfate ions that arise from the acid treatment. The overlapping of these bands makes it challenging to quantify the phosphate signals attributed to mineral content accurately. Without a clear resolution of these bands, isolating the contributions from mineral phases and assessing the mineral-to-matrix ratio reliably are difficult. The amide III band, around 1230–1300 cm^−1^, can partially overlap with phosphate and sulfate bands in the FTIR spectra. The presence of multiple overlapping bands diminishes the distinctiveness of each band, making it challenging to determine their contributions accurately. The interplay between these bands means that any change in one can affect the perceived intensity or area of the others, leading to potential inaccuracies in quantitative assessments.

### 2.3. LIBS and AAS Analysis

As the major component of bone tissue, calcium is one of the key elements of interest when analyzing bones with LIBS. The altered chemical composition of the bone and plasma conditions are reflected in the plasma’s intensity ratio of ionic and atomic calcium lines, Ca II/Ca I.

**Figure 5:**
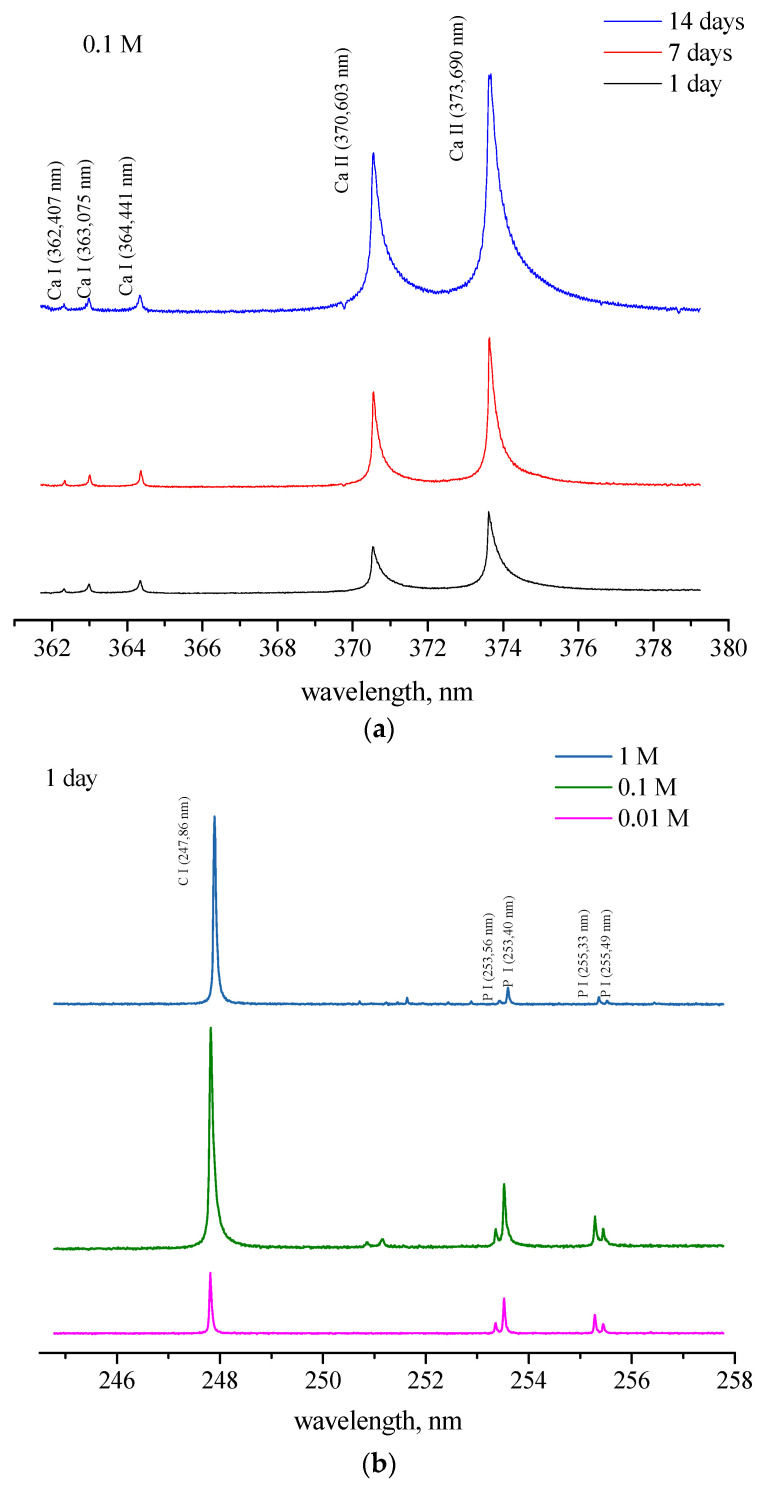
LIBS spectra of pig shoulder bone: Ca II and Ca I lines (0.1 M, different days) (**a**) and P I and C I lines ((**b**), day 1, different concentrations).

The formation of calcium sulfate on the bone surface influences the plasma’s local temperature and electron density and, consequently, the ionization state of calcium. The degree of ionization of calcium atoms in a plasma induced on a solid bone surface is an indicator of the temperature of the plasma and, indirectly, of the surface hardness of the bone, i.e., the mineral density of the bone [43,44]. To avoid self-absorption of calcium resonant lines, the ionic line at 364.441 nm (Ca II) and atomic line at 370.603 nm (Ca I) are used (Figure 5a) [45].

**Figure 6:**
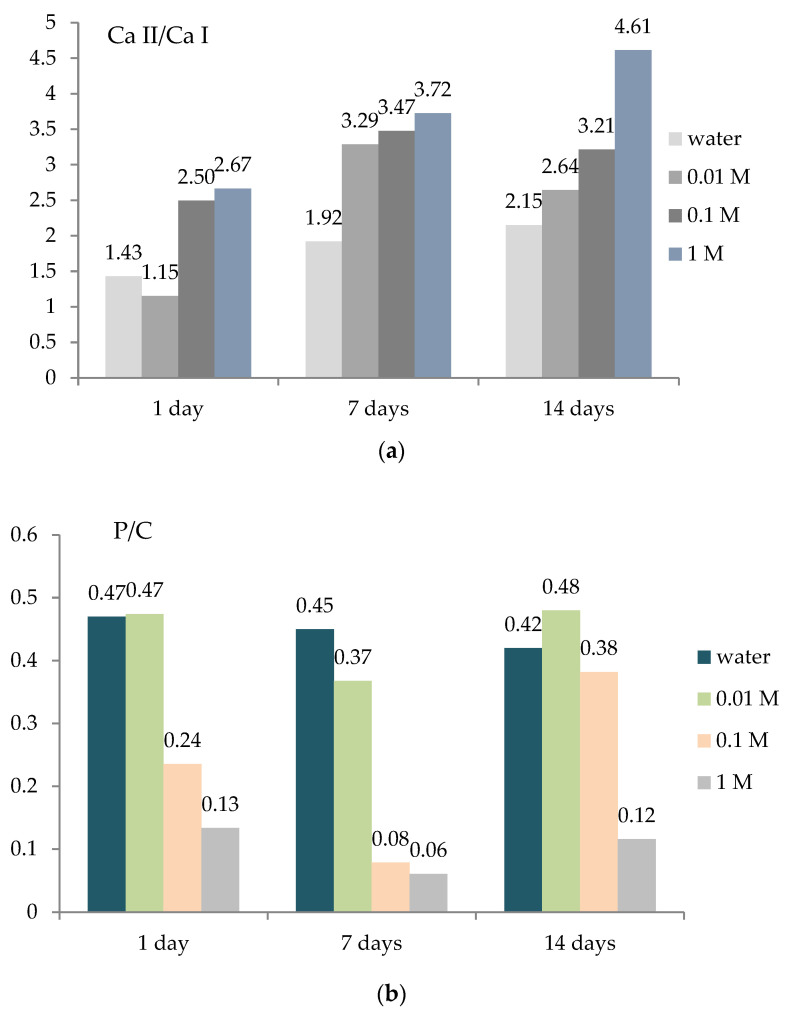
Intensity ratio of Ca II/Ca I lines (364.441/370.603 nm) (**a**) and P/C I lines (247.86/255.33 nm) (**b**) obtained from LIBS spectra.

The bar diagram in Figure 6a shows the intensity ratio of Ca II/Ca I lines obtained from LIBS spectra. It is evident that in the reference sample, the ratio slowly increases with time (1.41, 1.92, and 2.15), indicating that water can gradually dissolve minerals, including calcium, over time. If each period of acid action is analyzed, it can be seen that the ratio increases with increasing acid concentration (1.15–2.67 (day 1), 3.29–3.72 (day 7), and 2.64–4.61 (day 14)), which indicates a hardening of the bone surface as a result of the formation of calcium sulfate. The intensity ratios of phosphorous-to-carbon lines (P I/C I; 247.86 and 255.33 nm, respectively), obtained from LIBS spectra (Figure 5b), are used to monitor the relationship between the mineral and organic matrix. Those lines are selected based on the assumption that carbon and phosphorus experience similar excitation conditions, i.e., ionization temperatures around 250 nm. The results show that the reference sample’s mineral bone matrix content does not change significantly with time. A slight decrease in the P/C ratio (0.47, 0.45, and 0.42) indicates slow phosphorus leaching over time, which may alter the bone’s mineral content. The results suggest that the proportion of the mineralized part of the bone, observed by individual days, decreases with increasing acid concentration (0.47–0.13 (day 1); 0.37–0.06 (day 7); and 0.48–0.12 (day 14)) (Figure 6b). However, when comparing the results obtained for all days, this ratio is the highest after 14 days of acid action for all concentrations, followed by day 7 and day 1 (day 14 > day 7 > day 1) (Figure 6b).

**Figure 7:**
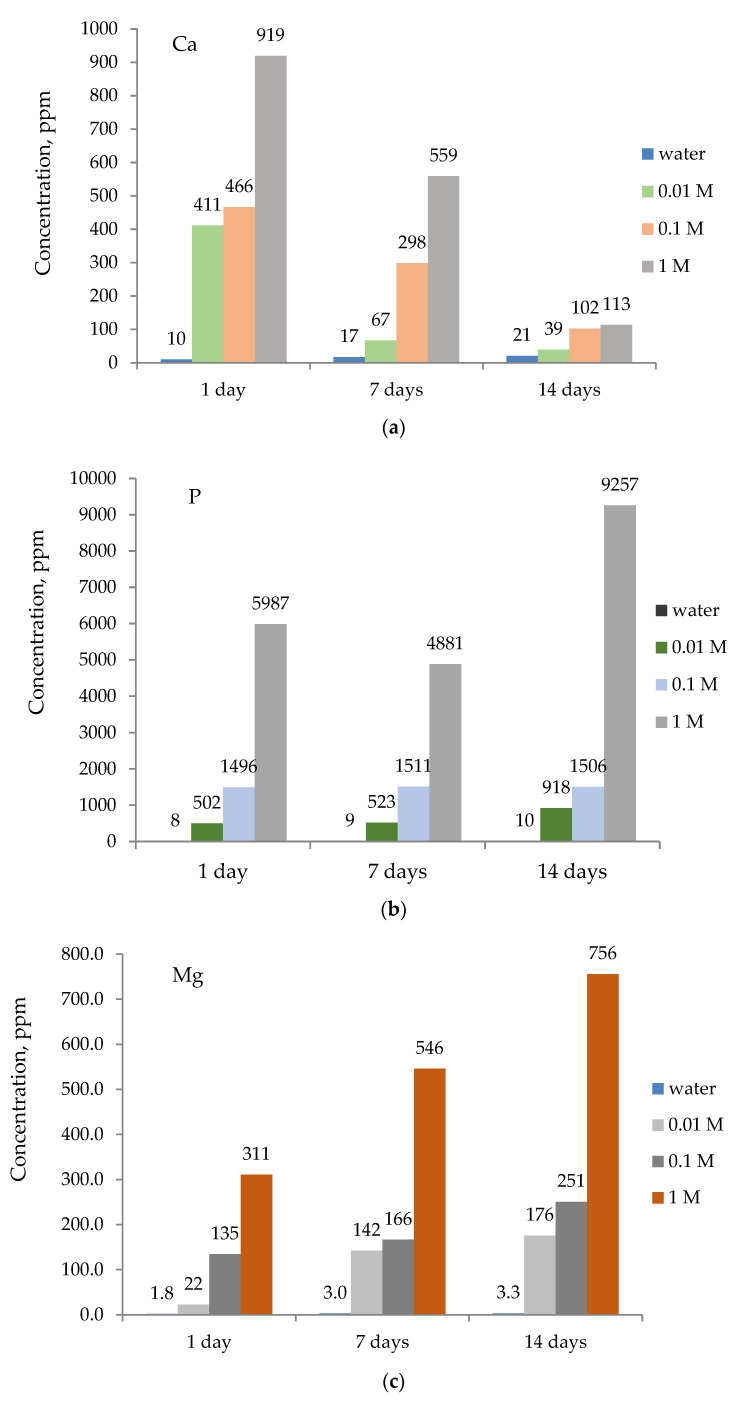
Concentration of calcium (**a**), phosphorous (**b**), and magnesium (**c**) in remaining solutions obtained by AAS.

Calcium, phosphorous, and magnesium contents in the solution, left after the bone removal, were estimated by AAS (Figure 7). The results show the high efficiency of sulfuric acid in bone demineralization. The calcium level in the reference solution shows a slight increase (10–21 ppm), which can be considered as a consequence of the slow leakage of calcium from the bone due to the action of water (Figure 7a). In contrast to the reference solution, as the bone surface becomes harder with prolonged acid action and increasing acid concentration due to the deposition of calcium sulfate, the calcium concentration in the solution decreases (411–39 ppm (0.01 M), 466–102 ppm (0.1 M), and 919–113 ppm (1 M)) (Figure 7a). Moreover, as the organic matrix of the bone is broken down, mineral components are also removed, and phosphate ions (PO_4_^3−^) are released into the solution. Figure 7b shows the change in phosphorus concentration in the solutions. In the reference sample, the phosphate content slightly increases (8–10 ppm) but in a much smaller amount than that of acid solutions.

In acid solutions by increasing acid concentration, the concentration of phosphorus in the solution also increases (502–4987 ppm (day 1); 523–5881 ppm (day 7)), reaching the highest value after 14 days (918–9257 ppm). After 14 days, the phosphorus concentration in the reference solution is almost a thousand times smaller than in the acid solution after the same period of time. The same trend follows the changes in magnesium concentration. In reference solution, the amount of magnesium is several hundreds of times less (1.8–3.3 ppm) compared to acid solutions (22–311 ppm (day 1), 142–546 ppm (day 7), and 176–756 ppm (day 14)) (Figure 7c), which indicates a significantly more efficient demineralization in an acidic environment. The LIBS and AAS analysis results can be considered very significant because they indicate a change in the mineral composition of the bone, which is both time- and concentration-dependent and, to our knowledge, is presented for the first time in this way.

## 3. Materials and Methods

### 3.1. Chemicals and Bone Samples

Sulfuric acid, p.a. grade purity, was purchased from Merck, Darmstadt Germany. The demineralization of pig shoulder bone was monitored in 0.01, 0.1, and 1.0 M aqueous sulfuric acid solutions. Pig shoulder bone, obtained from a butcher shop, was degreased in 70% *v*/*v* ethanol and later washed with distilled water. The sample was stored at 4 °C. Before being treated with sulfuric acid, the bone samples were all obtained by cutting pieces of the same bone with approximately the same mass (5 g), color, and visual structure. Bone samples kept in water for the same immersion period were taken as reference samples.

Each sample was placed in approximately 30 cm^3^ of acid solutions of different concentrations and left for 1, 7, and 14 days. The Reagecon (Clare, Ireland) Calcium/Magnesium/Phosphorous Standards for Atomic Absorption (AAS), 1000 μg/mL (1000 ppm) in 0.5 M nitric acid, were used as calibration standard solutions for the quantitative analysis of the elements of interest (Ca, Mg, and P).

### 3.2. ATR-FTIR Spectra

FTIR spectra of pig shoulder bone were recorded in the mid-IR region (4000–400 cm^−1^) using a Nicolet iS 20 FT-IR Spectrometer (Thermo Fisher Scientific, Waltham, MA USA) operating in the ATR mode. All the measurements were performed with a monolithic diamond crystal. The spectral resolution was 4 cm^−1^, and the number of scans was 16. The samples were recorded multiple times, and the averaged spectra were discussed. The vibrational spectra were analyzed using a curve fitting analysis in PeakFit program 4.12 and Voigt spectral line profile. Deconvolution was performed in the amide I (1700–1600 cm^−1^) and ν_1_/ν_3_ (PO_4_^3−^) phosphate (1200–900 cm^−1^) domains. Preliminary band positions were acquired from the second derivative spectra. Quantitative changes in bone composition were calculated from integrated areas of the underlying bands in examined domains.

### 3.3. LIBS Spectra and Elemental Analysis

The LIBS setup was based on a compact air-cooled pulsed Nd:YAG laser (model LQ115, Solar LS, Minsk, Belarus), which operated at a wavelength of 1064 nm (first harmonic). The energy of the laser pulse was 90 mJ, the frequency was 1 Hz, and the duration of the pulse was 7 ns. The laser beam was focused on the target using a glass lens with a focal length of 80 mm focused on the surface of the target, giving a spot with a diameter of approximately 140 μm. The image of the laser-induced plasma was projected onto the entrance slit of the spectrograph using an achromatic quartz lens (UV-achromat) (Knight Optical, Kent, UK) of focal length 273 mm, in a ratio of 1:1. Spectral emission acquisition was performed from a plasma slice parallel to the target, 1 mm away from the target.

Carl Zeiss PGS2 spectrograph (Jena, Germany) with a flat diffraction grating (blaze at 280 nm, 650 groves per mm) equipped with a U2C-16H11850 (SOL Instruments, Augsburg, Germany) CCD camera, with enhanced sensitivity in the UV range (2048 × 64 chip; pixel size 14 × 14 μm). LIBS spectra were recorded from 15 points on the bone surface, and 5 spectra were collected from each spot. One laser shot was sent to each selected point of the sample to minimize the degree of ablation and to obtain the best possible image of the bone surface composition. The results for each element analyzed are presented as the mean value of all measurements to avoid the composition variations within a sample and obtain a comprehensive analysis.

The Carl Zeiss Jena EA 30 atomic absorption spectrometer (Jena, Germany) was used for quantitative elemental analysis of solutions left after bone removal.

## 4. Conclusions

Combined techniques provide a comprehensive understanding of the effects of sulfuric acid on pig bone. Monitoring changes in the organic and inorganic matrix of the bone provided insights into the extent of structural integrity and functional properties of bone and mineral dissolution caused by the treatment of sulfuric acid of different concentrations and times of action. The obtained results indicate the complex secondary structure of collagen and the presence of carbonyl compounds formed upon the fragmentation of polypeptides. The most noticeable changes in the structure of the amide I band were evident after the first 24 h of acid action. Collagen maturity index values reflected the disturbed balance between mature and immature cross-links in treated bones, showing “bone aging” caused by acid treatment. The extensive dataset presents opportunities for further interpretation by correlating the changes in spectral features with mechanical property assessments. For instance, integrating mechanical testing methodologies alongside spectroscopic analyses could provide a more comprehensive understanding of how relevant sub-band trends relate to bone functionality. In the future, structural integrity could be characterized using tensile and compressive strength measurements. At the same time, the changes in spectral areas could facilitate the development of predictive models that link collagen stability in varying acidic environments to mechanical performance.

Sulfuric acid treatment also significantly influenced the mineral component, hydroxyapatite, of the bone. FTIR spectra in the ν_1_/ν_3_ (PO_4_^3−^) domain reveal the noticeable changes in intensity, width, structure, and position of the band assigned to ν_1_/ν_3_ (PO_4_^3−^) modes. The complex structure of bands in both the amide I and the ν_1_/ν_3_ (PO_4_^3−^) regions made it impossible to reliably establish quantitative relationships, like the mineral-to-matrix ratio, for assessing chemically induced bone alterations based on FTIR-ATR spectral data. This stems partly from the fragmentation of polypeptides, the mineral dissolution, the overlapping of phosphate and sulfate bands (from formed calcium sulfate), and even the amide III band. As a result, the ability to quantify the mineral-to-matrix ratio is compromised, leading to potential inaccuracies in understanding the structural and compositional changes in the bone matrix. The interactions and interferences in this spectral region highlight the need for careful future analysis and potentially the application of alternative methods or computational approaches to enhance interpretative clarity in such studies.

The intensity ratio of Ca II/Ca I lines indirectly reflected the mineral density and hardness of the bone. An increase in the Ca II/Ca I ratio with acid concentration and duration of acid action indicated hardening of the bone surface due to the formation of calcium sulfate.

The results of the AAS analysis of solutions left after bone removal show that the calcium, phosphorous, and magnesium contents change with time and acid concentration. As the bone surface becomes harder due to calcium sulfate formation, calcium concentration in the solutions drops eight times from the first to the fourteenth days of acid treatment (1 M solutions). Conversely, the concentration of phosphorus and magnesium in acid solutions increased 1.8 and 2.5 times, respectively, from the first to the fourteenth days of acid treatment (1 M solutions). In the reference solution, the concentration of all elements increases with time. Still, this increase is significantly smaller compared to the effect of sulfuric acid, almost a thousand times smaller, in the case of phosphorus, which indicates the pronounced demineralization ability of sulfuric acid.

As for real-world scenarios, especially in forensic and archaeological contexts, the complex interactions observed in pig bone’s organic and inorganic matrix under acidic conditions could aid forensic scientists and archaeologists in understanding how environmental factors and those caused by human action contribute to bone degradation. In this context, the changes in structural integrity, particularly the alterations in collagen secondary structures and mineral content, reflect how chemical treatments can affect skeletal remains. Such knowledge is crucial when examining real forensic cases or archaeological sites where the preservation state of bones may be compromised due to exposure to acids or other chemicals. In this case, the results from pig bone studies align closely with human studies; parallels regarding the rates of decomposition, structural changes, and mineral loss could be drawn, allowing for targeted forensic analyses to assess the condition and history of human remains. For instance, establishing a baseline for collagen stability and mineral density in chemically treated bones using methodologies such as FTIR and AASs could aid in identifying whether remains have been subjected to specific chemical treatments.

Future extensions of this work could address the effect of more chemicals and factors, like environmental conditions relevant to forensic or archaeological contexts, and the eventual comparison of results with results obtained on actual archeological/forensic samples. Also, other advanced spectroscopic techniques, like Raman spectroscopy, could enhance the depth of the analysis.

## Figures and Tables

**Table 1:** The results of the curve fitting analysis of the spectra of pig shoulder bone in the 1700–1600 cm^−1^ IR region (0.01 M solution).

Immersion Period (days)	Position of the Underlying Bands (cm^−1^)	Integrated Area (%)	Assignment of the Underlying Bands in the Amide I Region [32,33,34,35,36]
0.01 M H_2_SO_4_			
1	1622	7.8	β-turn
	1637	6.6	Triple helix
	1651	7.3	α helix
	1662	8.6	3_10_ helix; Pyridinoline collagen cross-links
	1673	7.9	Arginine side chain
	1692	12.5	DHLNL and HLNL cross-links
	1712	27.5	Glutamic acid side chain
	1735	21.8	Carbonyl vibrations
7	1610	18.7	β-turn
	1636	35.0	Triple helix
	1663	24.3	3_10_ helix; Pyridinoline collagen cross-links
	1688	13.4	DHLNL and HLNL cross-links
	1728	8.6	Carbonyl vibrations
14	1617	29.3	β-turn
	1639	25.3	Triple helix
	1662	26.3	3_10_ helix; Pyridinoline collagen cross-links
	1689	12.6	DHLNL and HLNL cross-links
	1734	6.5	Carbonyl vibrations

## Data Availability

The original contributions presented in the study are included in the article/Supplementary Materials; further inquiries can be directed to the corresponding author/s.

## Supplementary Materials

The following supporting information can be downloaded at https://www.mdpi.com/article/10.3390/ijms252212250/s1.
