# Supplementary material for: From Structure to Strength: Analyzing the Impact of Sulfuric Acid on Pig Bone Demineralization Through FTIR, LIBS, and AAS
Source: Int J Mol Sci. 2024 Nov 14;25(22):12250. doi: 10.3390/ijms252212250 (PMC11594744; doi:10.3390/ijms252212250)
Supplement: Supplementary file 1 [file ijms-25-12250-s001.zip › ijms-3267956-supplementary.pdf]

Supplementary Material

From Structure to Strength: Analyzing the Impact of Sulfuric Acid on Pig Bone Demineralization through FTIR, LIBS, and AAS

Milica Marković[1], Miroslav Kuzmanović[1], Dragan Ranković[a], Danica Bajuk-Bogdanović[1], Aleksandra Šajić[1], Dušan Dimić[1*]

[1] University of Belgrade, Faculty of Physical Chemistry, Studentski trg 12-16, 11000 Belgrade, Serbia; : milica.markovic@ffh.bg.ac.rs (M.M.); miroslav@ffh.bg.ac.rs (M.K.); danabb@ffh.bg.ac.rs (D.B.B.); aleksandra.sajic@ffh.bg.ac.rs (A.Š.)
[2]University of Belgrade, Institute of Nuclear Sciences, Mike Petrovića Alasa 12-14, 11351 Vinča, Belgrade, Serbia, ranko@ffh.bg.ac.rs (D.R.)

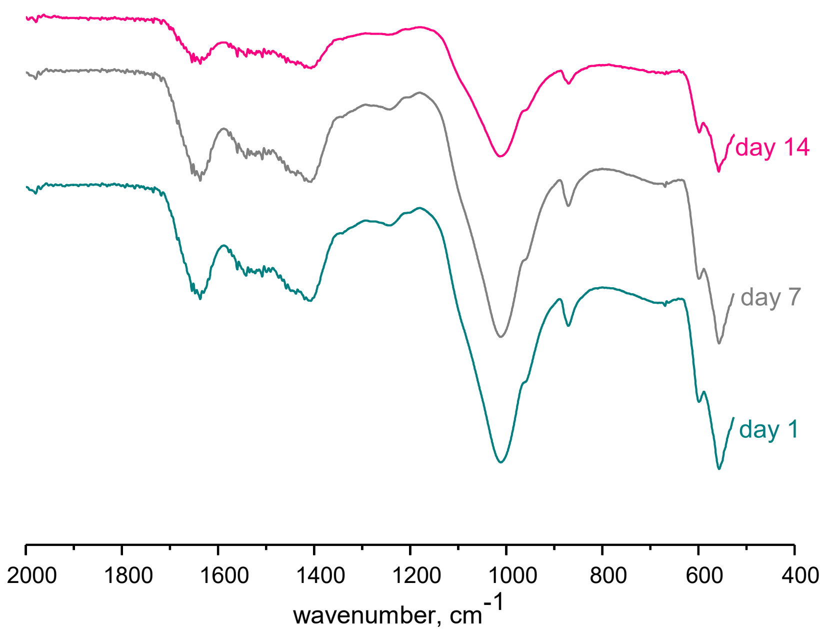

Figure S1. FTIR spectra of pig shoulder bone in water: immersion time, 1, 7, and 14 days.

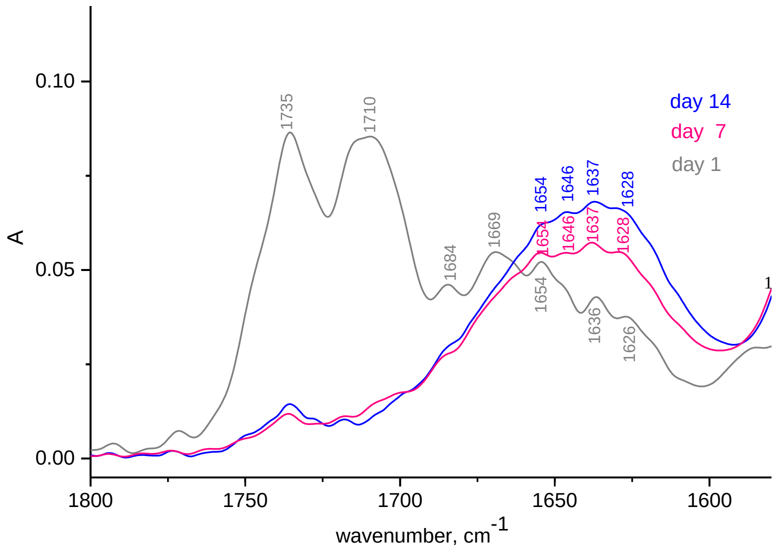

(a)

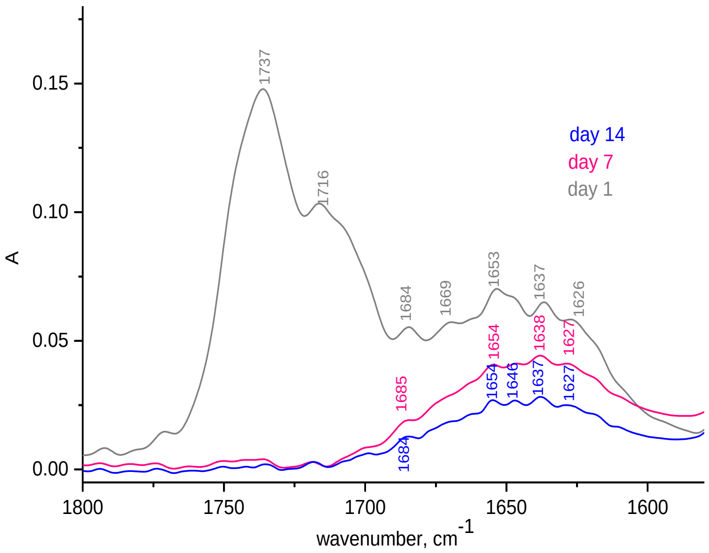

(b)

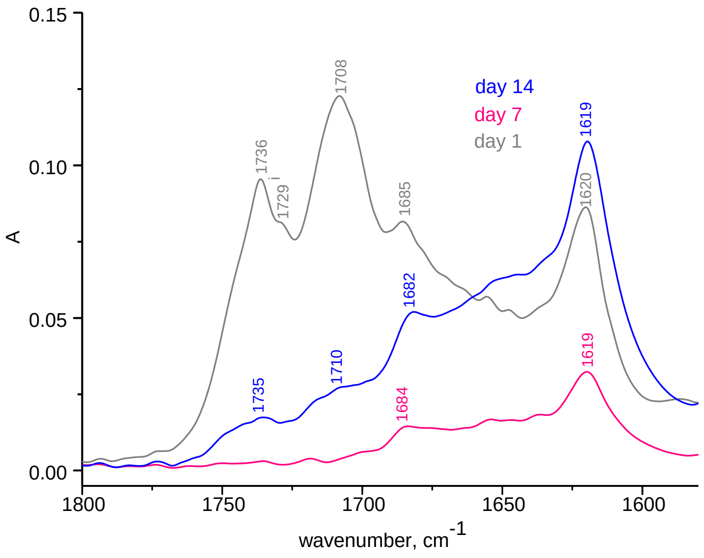

(c)

Figure S2. Comparative display of the 1800-1600 cm^{-1} FTIR region of pig shoulder bone in 0.01 M (a), 0.1 M (b), and 1.0 M (c) sulfuric acid (immersion time, 1, 7, and 14 days).

Table S1. The results of a curve fitting analysis of the spectra of the pig shoulder bone in the 1750-1600 cm^{-1} IR region (0.1 and 1 M solutions).

Immersion period (days)	Position of the underlying bands (cm^{-1})	Integrated area (%)	Assignment of the underlying bands in the Amide I region [32-36]
**water**			
**1**	1621	20.8	β-turn
	1640	38.5	Triple helix
	1661	23.1	3_{10} helix; Pyridinoline collagen cross-links
	1686	17.6	DHLNL and HLNL cross-links
**7**	1621	20.2	β-turn
	1640	38.0	Triple helix
	1661	21.6	3_{10} helix; Pyridinoline collagen cross-links
	1686	20.2	DHLNL and HLNL cross-links
**14**	1621	19.2	β-turn
	1640	35.5	Triple helix
	1661	20.0	3_{10} helix; Pyridinoline collagen cross-links
	1686	25.3	DHLNL and HLNL cross-links
**0.1 M H_2SO_4**			
**1**	1619	7.8	β-turn
	1640	12.3	Triple helix
	1662	13.0	3_{10} helix; Pyridinoline collagen cross-links
	1689	12.5	DHLNL and HLNL cross-links
	1712	18.8	Glutamic acid side chain
	1735	24.3	Carbonyl vibrations
	1748	11.3	Carbonyl vibrations
**7**	1601	12.7	Tyrosine side chain
	1619	18.1	β-turn
	1638	29.6	Triple helix
	1660	26.1	3_{10} helix; Pyridinoline collagen cross-links
	1681	9.6	Deoxypyridinoline cross-link, parallel β-sheet
	1699	3.9	Anti-parallel β-sheet
**14**	1602	12.4	Tyrosine side chain
	1622	20.3	β-turn
	1641	29.2	Triple helix
	1663	24.0	3_{10} helix; Pyridinoline collagen cross-links
	1687	12.3	DHLNL and HLNL cross-links
	1704	1.8	Glutamic acid side chain
**1.0 M H_2SO_4**			
**1**	1603	4.3	Tyrosine side chain
	1620	9.7	β-turn
	1635	9.9	Triple helix
	1661	15.9	3_{10} helix; Pyridinoline collagen cross-links
	1690	16.4	DHLNL and HLNL cross-links
	1710	20.4	Glutamic acid side chain
	1737	23.4	Carbonyl vibrations
**7**	1604	10.0	Tyrosine side chain
	1620	29.7	β-turn
	1640	20.6	Triple helix
	1662	19.8	3_{10} helix; Pyridinoline collagen cross-links
	1686	12.0	DHLNL and HLNL cross-links
	1710	7.9	Glutamic acid side chain
**14**	1602	11.2	Tyrosine side chain
	1620	19.7	β-turn
	1639	21.3	Triple helix
	1662	22.8	3_{10} helix; Pyridinoline collagen cross-links
	1687	12.0	DHLNL and HLNL cross-links
	1710	5.6	Glutamic acid side chain
	1735	7.4	Carbonyl vibrations

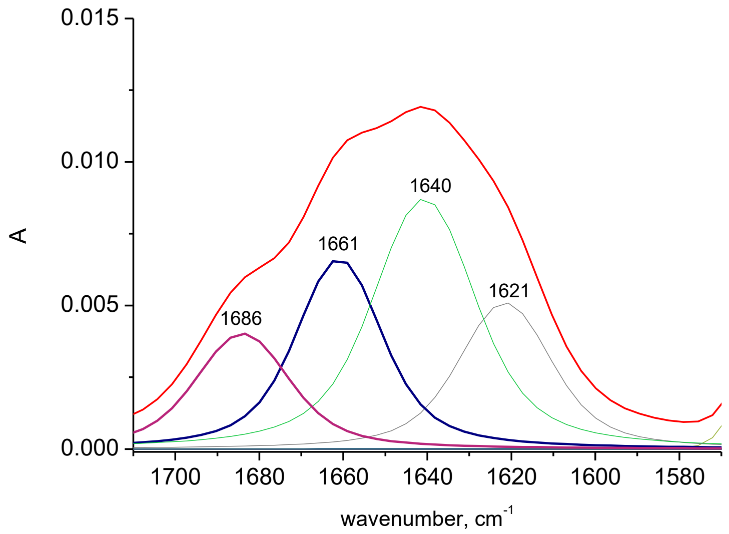

(a)

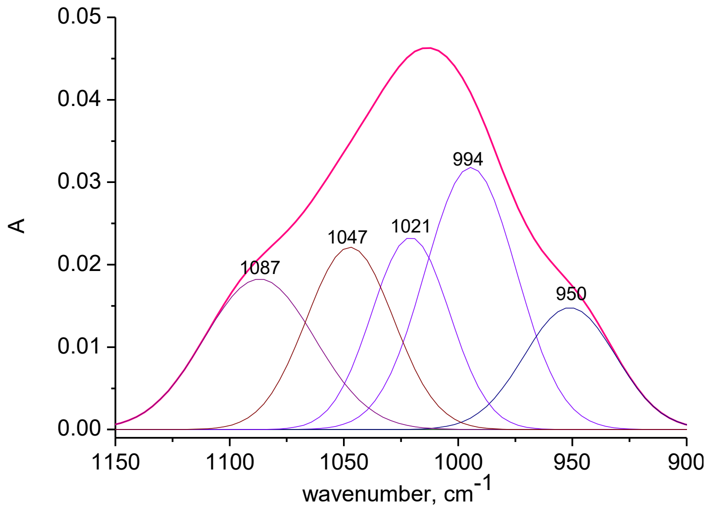

(b)

Figure S3. The results of a curve fitting analysis in the 1700-1600 cm^{-1} (a) and 1200-900 cm^{-1} IR regions (b) of pig bone in a reference spectrum: immersion time, 1 day.

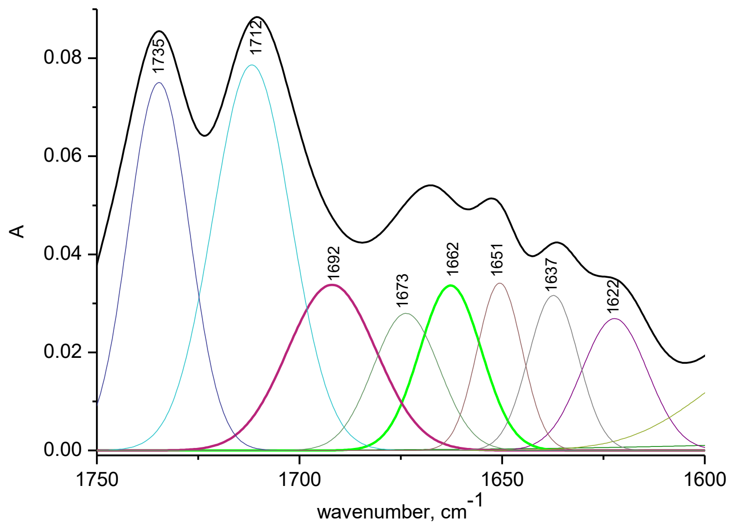

(a)

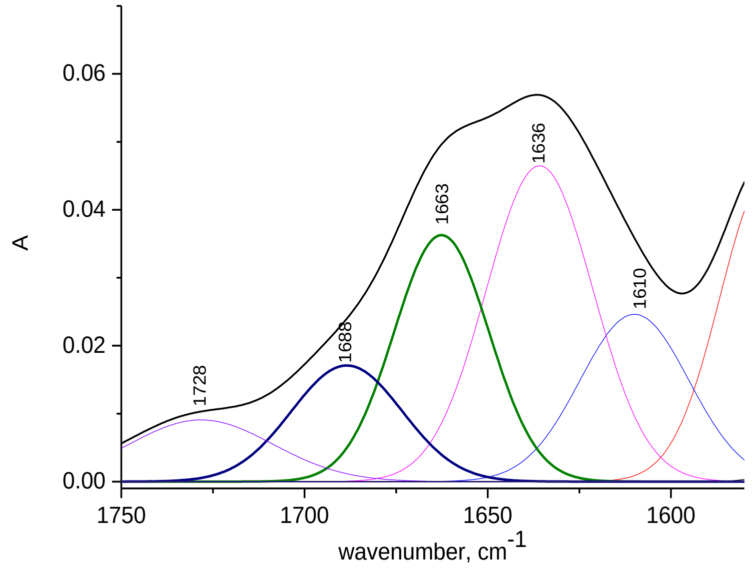

(b)

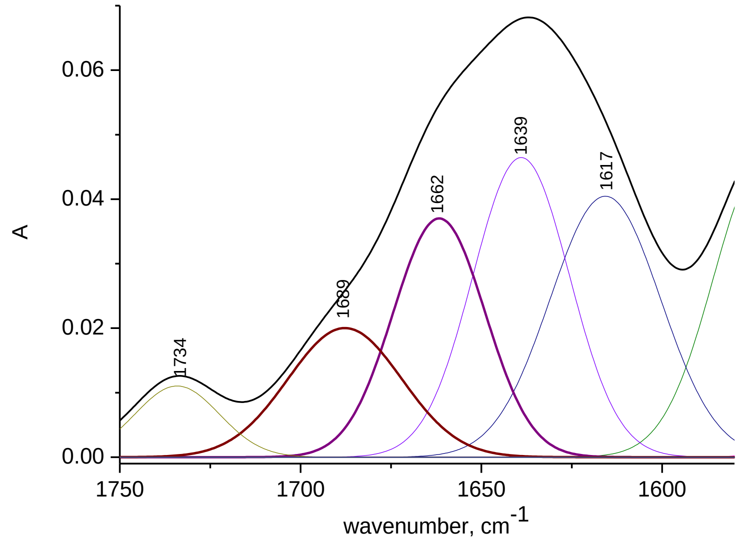

(c)

Figure S4. The results of a curve fitting analysis in the 1800-1600 cm^{-1} IR region of pig bone in 0.01 M sulfuric acid: immersion time, 1 (a), 7 (b), and 14 (c) days.

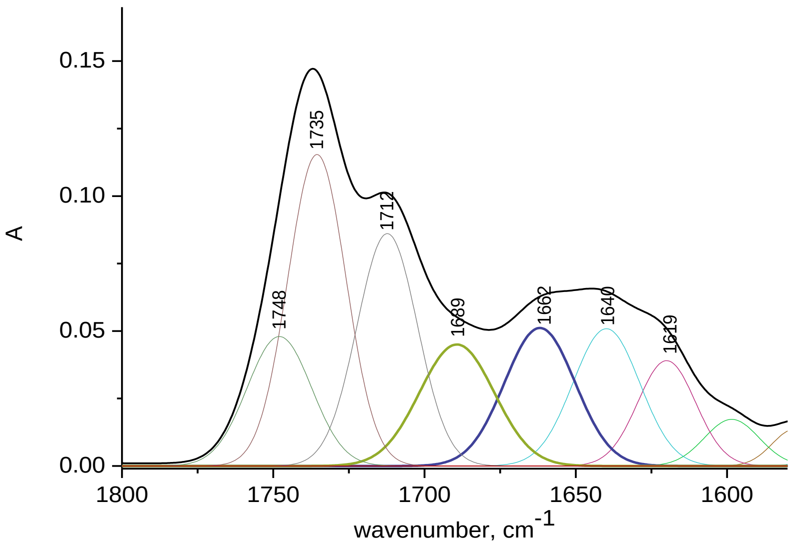

(a)

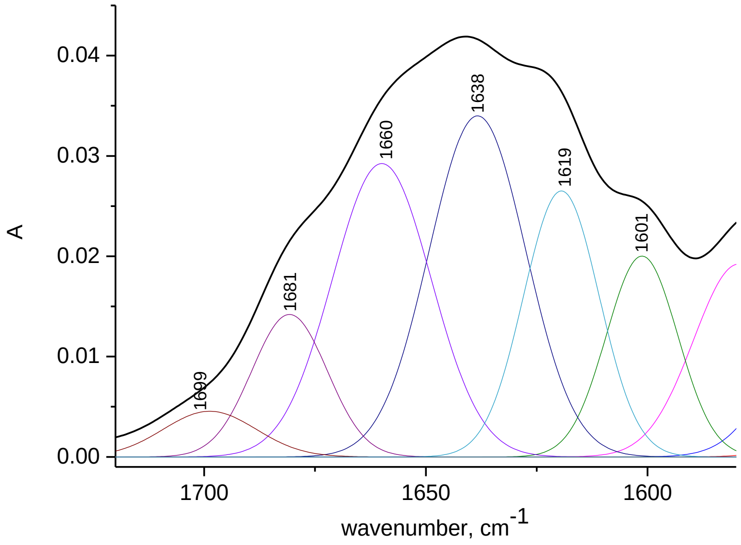

(b)

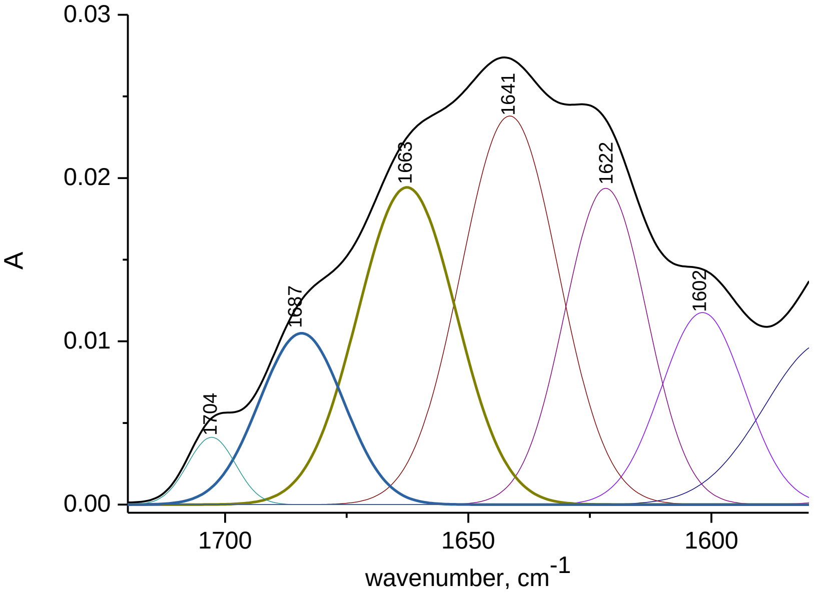

(c)

Figure S5. The results of a curve fitting analysis in the 1800-1600 cm^{-1} IR region of pig bone in 0.1 M sulfuric acid: immersion time, 1 (a), 7 (b), and 14 (c) days.

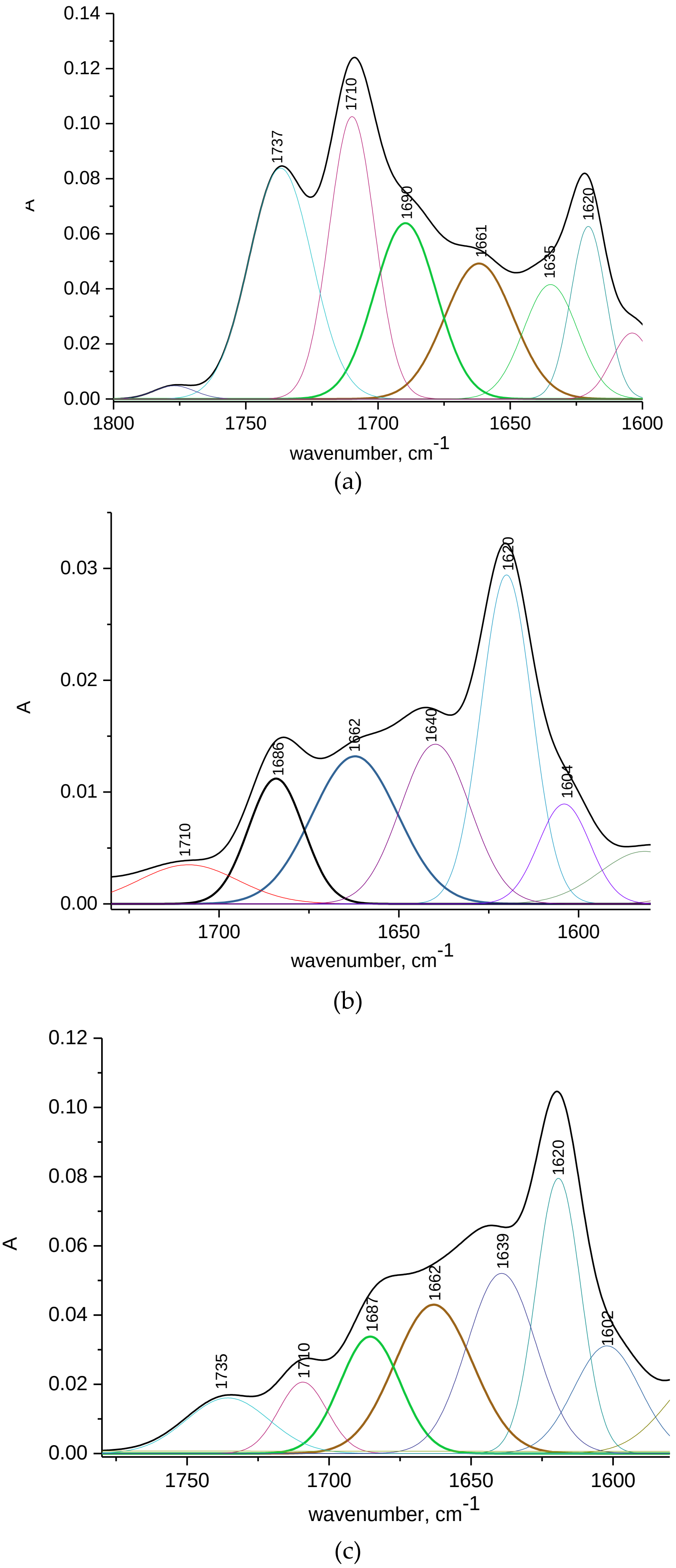

Figure S6. The results of a curve fitting analysis in the 1800-1600 cm^{-1} IR region of pig bone in 1.0 M sulfuric acid: immersion time, 1 (a), 7 (b), and 14 (c) days.

Table S2. The results of the curve fitting analysis of the spectra of pig shoulder bone in the 1300-900 cm^{-1} IR region (0.01, 0.1, and 1 M solutions).

Period of immersion	Position of the underlying bands (cm^{-1})	Integrated area (%)	Assignment of the underlying bands In the $\nu_1/\nu_3(PO_4^{3-})$ region [14-19]
		**water**	
1	951	13.5	HPO_4^{2-}-PO symmetric stretch
	994	28.9	$\nu_1(PO_4^{3-})$ in apatitic environment
	1021	18.2	$\nu_3(PO_4^{3-})$ domain, non-stoichiometric HA containing vacancies, HPO_4^{2-} and/or CO_3^{2-}
	1046	19.3	type B carbonate apatites; hydroxyapatite
	1087	20.1	$\nu_3(PO_4^{3-})$ in stoichiometric apatites
7	953	25.8	HPO_4^{2-}-PO symmetric stretch
	994	20.3	$\nu_1(PO_4^{3-})$ in apatitic environment
	1022	24.9	$\nu_3(PO_4^{3-})$ domain, non-stoichiometric HA containing vacancies, HPO_4^{2-} and/or CO_3^{2-}
	1055	17.1	$\nu_3(T_2)$ vibrational modes of apatite
	1093	11.9	$\nu_3(PO_4^{3-})$ domain in stoichiometric apatites
14	953	26.1	HPO_4^{2-}-PO symmetric stretch
	993	19.7	$\nu_1(PO_4^{3-})$ in apatitic environment
	1021	26.5	$\nu_3(PO_4^{3-})$ domain, non-stoichiometric HA containing vacancies, HPO_4^{2-} and/or CO_3^{2-}
	1053	15.3	$\nu_3(T_2)$ vibrational modes of apatite
	1089	12.4	$\nu_3(PO_4^{3-})$ in stoichiometric apatites
		**0.01 M H_2SO_4**	
1	943	1.6	HPO_4^{2-}-PO symmetric stretch
	965	4.0	$\nu_1(PO_4^{3-})$ symmetric stretch
	987	4.8	PO_4^{3-} in apatitic environment
	1007	8.3	$\nu_3(PO_4^{3-})$ asymmetric stretch
	1029	13.3	$\nu_3(PO_4^{3-})$ in stoichiometric apatites
	1054	9.2	$\nu_3(T_2)$ vibrational modes of apatite
	1079	4.2	$\nu_3(T_2)$ vibrational modes of apatite
	1098	4.5	$\nu_3(PO_4^{3-})$ domain, stoichiometric apatites
	1114	1.8	$\nu_3(PO_4^{3-})$ domain, poorly crystalline apatites
	1163	6.7	[1]
	1180	10.8	[1]
	1198	7.3	[1]
	1224	7.6	[1]
	1246	8.8	[1]
	1269	5.0	[1]
	1289	2.8	[1]
7	935	4.0	HPO_4^{2-}-PO symmetric stretch
	961	11.4	$\nu_1(PO_4^{3-})$ symmetric stretch
	993	18.2	$\nu_1(PO_4^{3-})$ in apatitic environment
	1020	26.5	$\nu_3(PO_4^{3-})$ domain, non-stoichiometric HA containing vacancies, HPO_4^{2-} and/or CO_3^{2-}
	1050	18.3	$\nu_3(T_2)$ vibrational modes of apatite
	1078	10.7	$\nu_3(T_2)$ vibrational modes of apatite
	1105	10.9	$\nu_3(PO_4^{3-})$ domain, poorly crystalline apatites
14	937	5.4	HPO_4^{2-}-PO symmetric stretch
	965	11.4	$\nu_1(PO_4^{3-})$ symmetric stretch
	995	19.6	PO_4^{3-} in apatitic environment
	1021	23.0	$\nu_3(PO_4^{3-})$ domain, non-stoichiometric HA containing vacancies, HPO_4^{2-} and/or CO_3^{2-}
	1049	15.6	$\nu_3(T_2)$ vibrational modes of apatite

	1078	12.6	$\nu_3(T_2)$ vibrational modes of apatite
	1107	7.1	$\nu_3(PO_4^{3-})$ domain, poorly crystalline apatites
	1160	5.3	[1]
	**0.1 M H_2SO_4**		
**1**	921	2.6	HPO_4^{2-}-PO symmetric stretch
	961	7.4	$\nu_1(PO_4^{3-})$ symmetric stretch
	991	7.6	PO_4^{3-} in apatitic environment
	1020	12.7	$\nu_3(PO_4^{3-})$ domain, non-stoichiometric HA containing vacancies, HPO_4^{2-} and/or CO_3^{2-}
	1055	11.7	$\nu_3(T_2)$ vibrational modes of apatite
	1089	10.8	$\nu_3(PO_4^{3-})$ domain in stoichiometric apatites
	1113	8.2	[1]
	1146	10.3	[1]
	1173	10.5	[1]
	1196	5.5	[1]
	1221	4.1	[1]
	1244	4.6	[1]
	1266	2.5	[1]
	1287	1.5	[1]
**7**	920	7.1	HPO_4^{2-}-PO symmetric stretch
	958	13.5	$\nu_1(PO_4^{3-})$ symmetric stretch
	994	21.7	PO_4^{3-} in apatitic environment
	1021	18.8	$\nu_3(PO_4^{3-})$ domain, non-stoichiometric HA containing vacancies, HPO_4^{2-} and/or CO_3^{2-}
	1051	18.3	$\nu_3(T_2)$ vibrational modes of apatite
	1086	14.7	$\nu_3(PO_4^{3-})$ domain in stoichiometric apatites
	1119	4.3	[1]
	1155	1.6	[1]
**14**	921	9.2	HPO_4^{2-}-PO symmetric stretch
	958	15.4	$\nu_1(PO_4^{3-})$ symmetric stretch
	991	22.4	PO_4^{3-} in apatitic environment
	1021	23.1	$\nu_3(PO_4^{3-})$ domain, non-stoichiometric HA containing vacancies, HPO_4^{2-} and/or CO_3^{2-}
	1057	17.0	$\nu_3(T_2)$ vibrational modes of apatite
	1097	10.9	$\nu_3(PO_4^{3-})$ domain in stoichiometric apatites
	1130	2.0	[1]
	**1.0 M H_2SO_4**		
**1**	921	2.0	HPO_4^{2-}-PO symmetric stretch
	965	5.0	$\nu_1(PO_4^{3-})$ symmetric stretch
	996	5.4	PO_4^{3-} in apatitic environment
	1021	7.8	$\nu_3(PO_4^{3-})$ domain, non-stoichiometric HA containing vacancies, HPO_4^{2-} and/or CO_3^{2-}
	1053	12.8	$\nu_3(T_2)$ vibrational modes of apatite
	1086	21.0	$\nu_3(PO_4^{3-})$ domain in stoichiometric apatites
	1110	20.4	$\nu_3(PO_4^{3-})$ domain, poorly crystalline apatites
	1136	18.3	[1]
	1164	7.3	[1]
**7**	921	3.9	HPO_4^{2-}-PO symmetric stretch
	960	6.4	$\nu_1(PO_4^{3-})$ symmetric stretch
	993	7.8	PO_4^{3-} in apatitic environment
	1021	10.7	$\nu_3(PO_4^{3-})$ domain, non-stoichiometric HA containing vacancies, HPO_4^{2-} and/or CO_3^{2-}

	1056	16.4	$\nu_3(T_2)$ vibrational modes of apatite
	1089	21.8	$\nu_3(PO_4^{3-})$ domain in stoichiometric apatites
	1114	14.8	$\nu_3(PO_4^{3-})$ domain, poorly crystalline apatites
	1139	12.5	[1]
	1164	5.7	[1]
**14**	930	1.9	HPO_4^{2-}-PO symmetric stretch
	961	5.9	$\nu_1(PO_4^{3-})$ symmetric stretch
	994	9.9	PO_4^{3-} in apatitic environment
	1022	8.5	$\nu_3(PO_4^{3-})$ domain, non-stoichiometric HA containing vacancies, HPO_4^{2-} and/or CO_3^{2-}
	1053	11.6	$\nu_3(T_2)$ vibrational modes of apatite
	1081	15.0	$\nu_3(T_2)$ vibrational modes of apatite
	1109	26.9	$\nu_3(PO_4^{3-})$ domain, poorly crystalline apatites
	1136	13.3	[1]
	1160	7.0	[1]

[1] Bands above 1110 cm^{-1} cannot be unambiguously assigned to the vibrations of the phosphate group due to sulfate bands (calcium sulfate formed as a result of phosphate dissolution) which also appear in the same region.

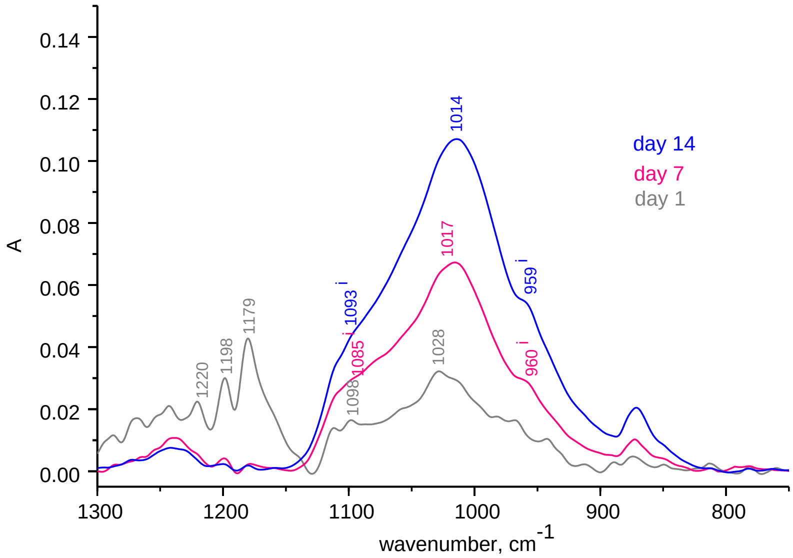

(a)

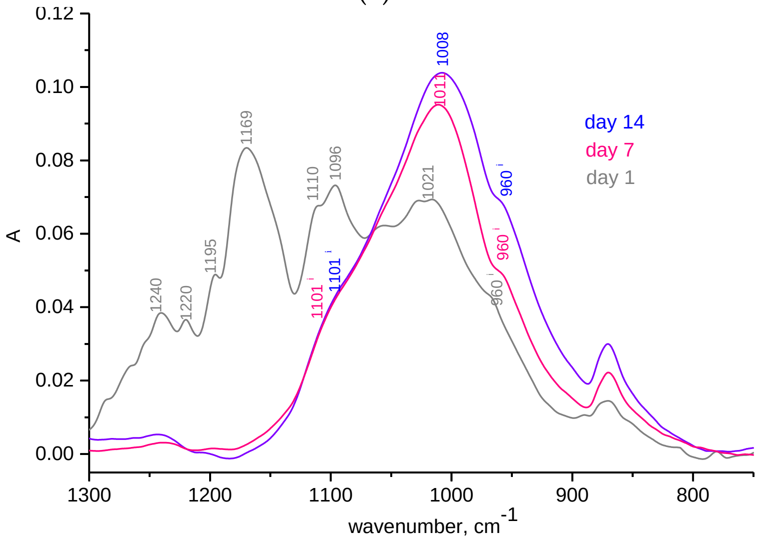

(b)

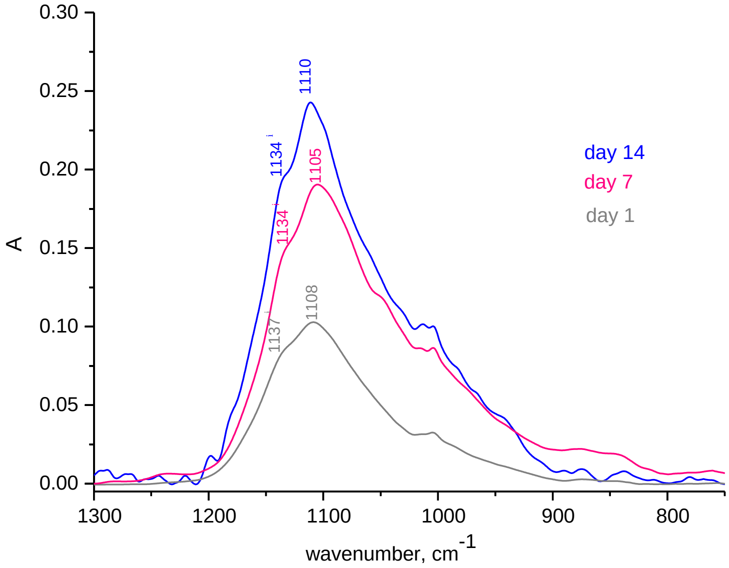

(c)

Figure S7. Comparative display of the 1300-900 cm^{-1} FTIR region of pig shoulder bone in 0.01 M (a), 0.1 M (b), and 1.0 M (c) sulfuric acid solutions (immersion time, 1, 7, and 14 days).

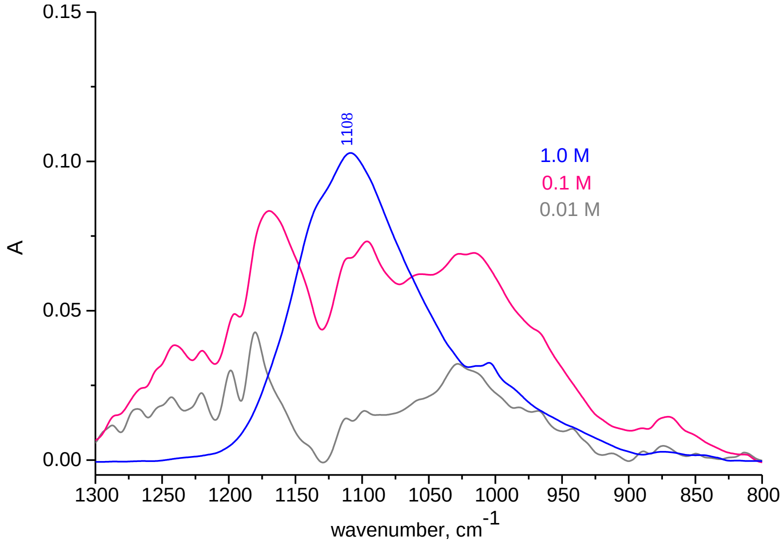

(a)

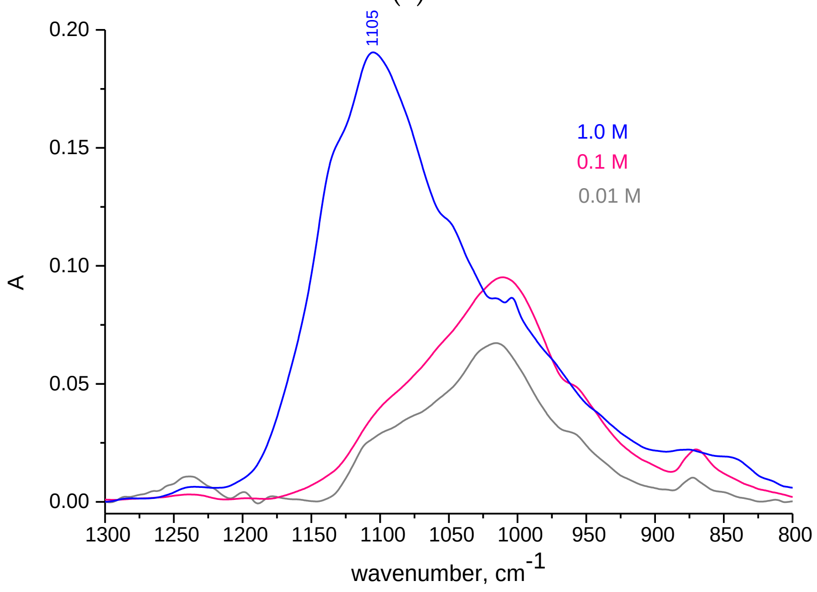

(b)

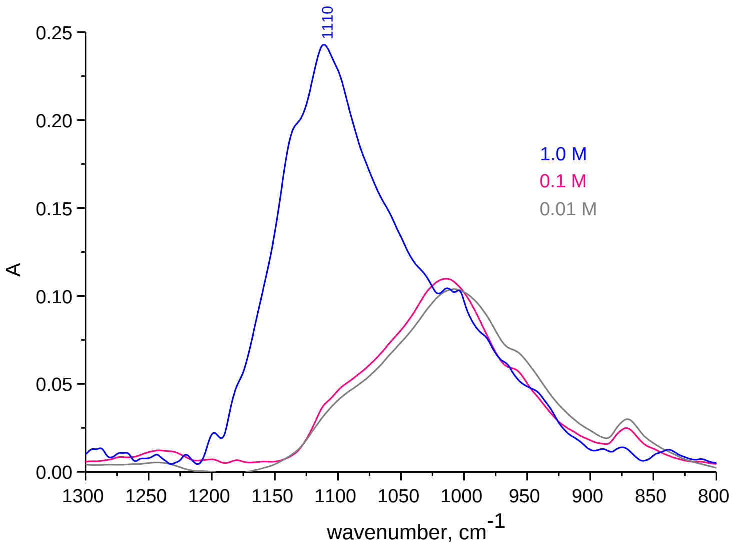

(c)

Figure S8. Comparative display of the 1300-900 cm^{-1} FTIR region of pig shoulder bone in sulfuric acid of different concentrations: immersion time, 1 (a), 7 (b), and 14 (c) days.

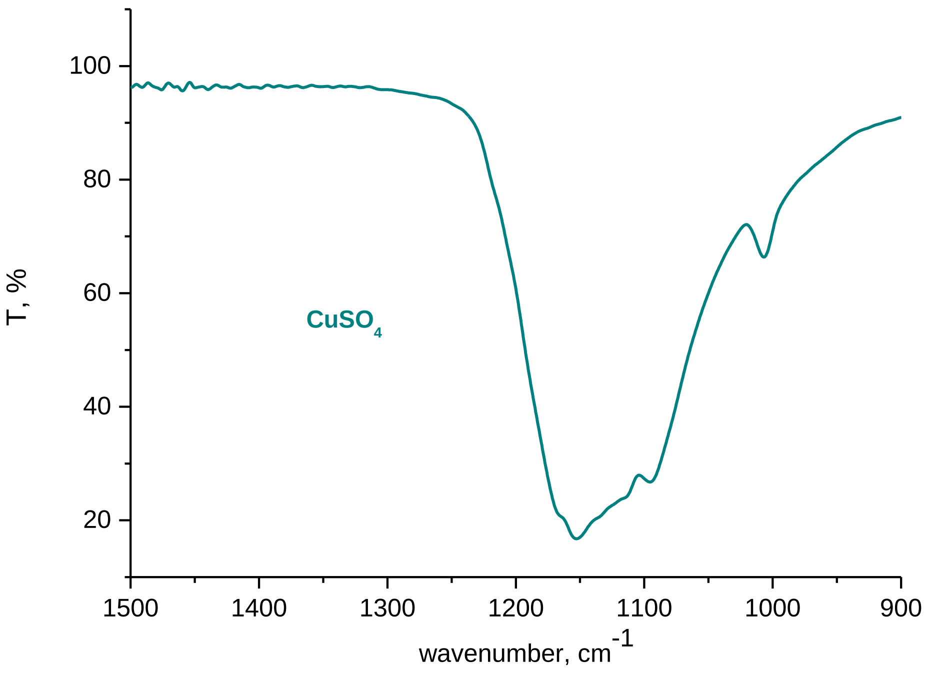

Figure S9. ATR-FTIR spectrum of calcium sulfate.

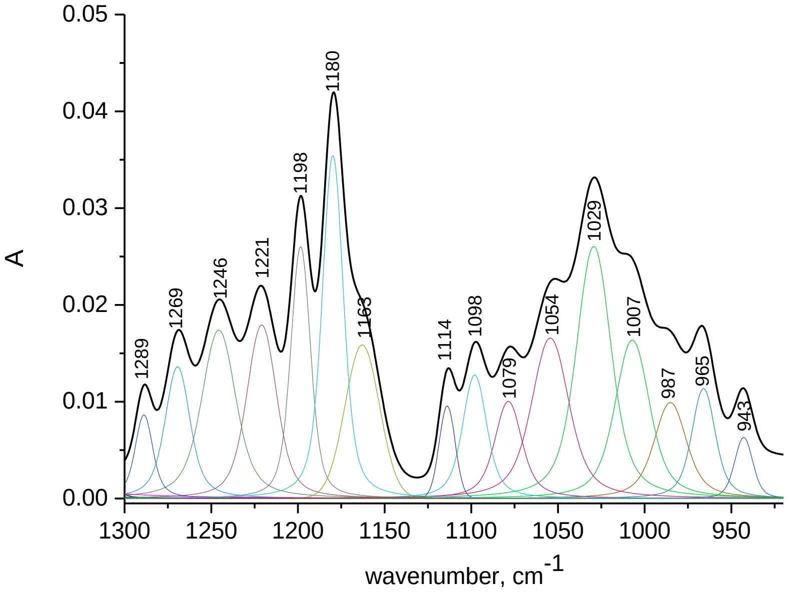

(a)

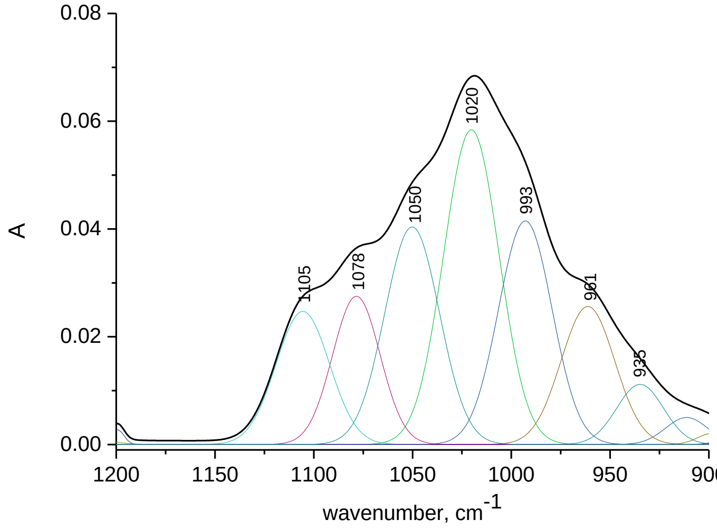

(b)

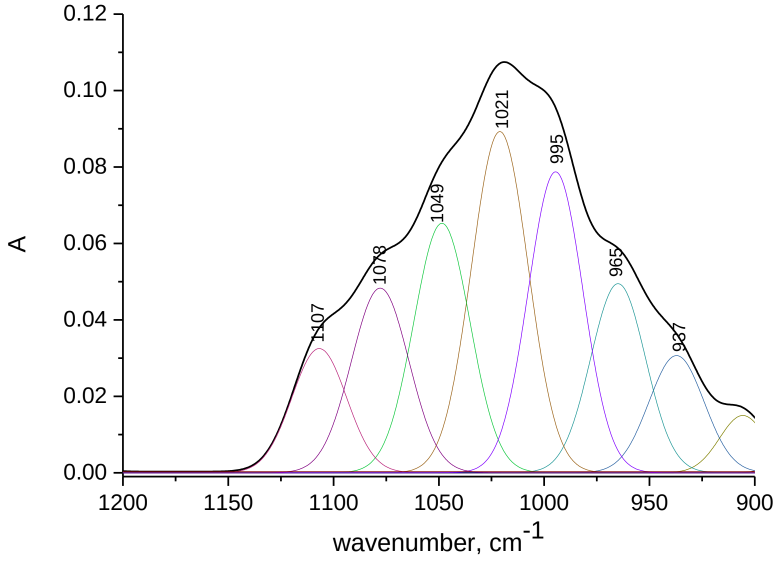

(c)

Figure S10. The results of the curve fitting analysis in the 1300-900 cm^{-1} IR region of pig bone in 0.01 M sulfuric acid: immersion time, 1 (a), 7 (b), and 14 (c) days.

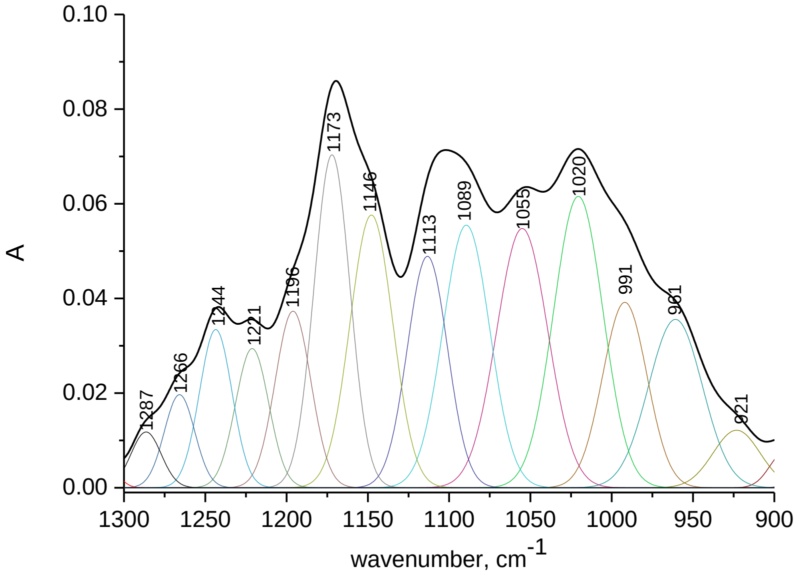

(a)

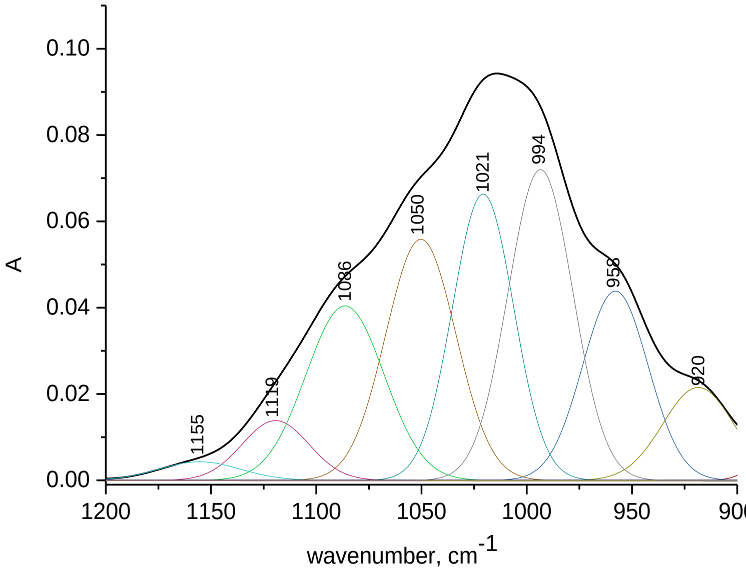

(b)

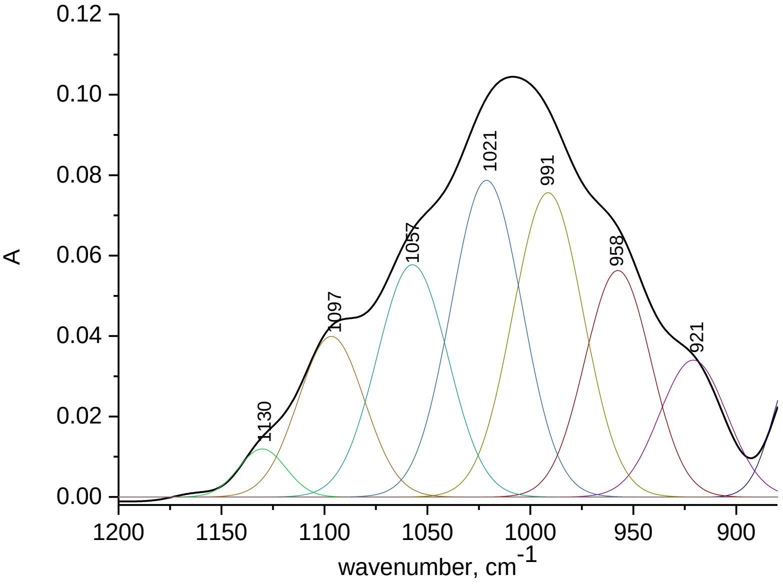

(c)

Figure S11. The results of the curve fitting analysis in the 1300-900 cm^{-1} IR region of pig bone in 0.1 M sulfuric acid: immersion time, 1 (a), 7 (b), and 14 (c) days.
